# Dysregulated NK-cell gene expression defines the enduring symptoms of long COVID-19

**DOI:** 10.3389/fimmu.2026.1720551

**Published:** 2026-03-09

**Authors:** Urvi Ray, Antje Schulze Selting, Roshan Priyarangana Perera, Zhiqi Yang, Vladislav Lysenkov, Siri Göpel, Michael Bitzer, Madhuri S. Salker, Stephan Ossowski, Olaf Riess, Nicolas Casadei, Yogesh Singh

**Affiliations:** 1 Institute of Medical Genetics and Applied Genomics, University of Tübingen, Tübingen, Germany; 2 Research Institute of Women’s Health, University of Tübingen, Tübingen, Germany; 3 Next Generation Sequencing Competence Center Tübingen (NCCT), University of Tübingen, Tübingen, Germany; 4 Department of Internal Medicine I, University Hospital Tübingen, Tübingen, Germany; 5 German Centre for Infection Research (DZIF), Braunschweig, Germany; 6 Women’s Health Research Institute, Vancouver, BC, Canada; 7 Department of Obstetrics and Gynecology, University of British Columbia, Vancouver, BC, Canada; 8 British Columbia (BC) Children’s Hospital Research Institute, Vancouver, BC, Canada

**Keywords:** cytokines, long COVID, NK cells, PBMCs, scRNA-seq, LTCS

## Abstract

**Introduction:**

Long-term COVID-19 syndrome (LTCS) or “long COVID” is a debilitating post-viral condition affecting approximately 2%–8% of individuals after SARS-CoV-2 infection. It manifests typically ≥3 months post-infection with symptoms persisting for at least 2 months, including fatigue, pulmonary dysfunction, and cognitive impairment, in the absence of alternative diagnoses. The biological mechanisms underlying LTCS remain poorly defined, yet emerging evidence implicates immune dysregulation.

**Methods:**

We profiled plasma antibodies and cytokines from healthy controls (HC, *N* = 66), convalescents (CONV, *N* = 24), and LTCS patients (*N* = 94), followed by multiparametric 14-color flow cytometry of PBMCs from HC (*N* = 9), CONV (*N* = 6), and LTCS (*N* = 23) participants. To gain mechanistic insight, we performed single-cell transcriptomic profiling (scRNA-seq) on PBMCs from HC (*N* = 8), CONV (*N* = 6), and LTCS (*N* = 32) individuals.

**Results:**

LTCS patients exhibited elevated anti-SARS-CoV-2 IgG (spike S1/RBD/N) titers compared with HC, but displayed significantly reduced systemic cytokine levels, including IFN-γ, TNF-α, IL-6, and IL-10. Flow cytometry revealed marked depletion of CD56^+^CD16^+^ NK cells and CD56^+^CD3^+^ NKT cells, accompanied by altered T-cell activation states. scRNA-seq confirmed NK type I cell loss and uncovered broad transcriptional reprogramming with upregulation of *PDCD4*, *CHD1*, *CXCR4*, and *SLC7A5* and downregulation of *TGFBR3*, *RIPOR2*, and *MBNL1*. Gene set enrichment analyses indicated activation of circadian and translational programs and suppression of olfactory receptor, neurotransmitter receptor, and GABA-gated ion-channel pathways. Functional assays validated reduced NK-cell inflammatory capacity in LTCS participants.

**Discussion:**

LTCS is characterized by systemic cytokine attenuation and a quantitative and functional NK-cell deficit coupled to neurosensory pathway suppression. These findings identify NK cells as key sentinels of LTCS pathophysiology and highlight an NK-centric neuroimmune axis as a promising target for biomarker discovery and therapeutic intervention.

## Graphical abstract

**Figure:**
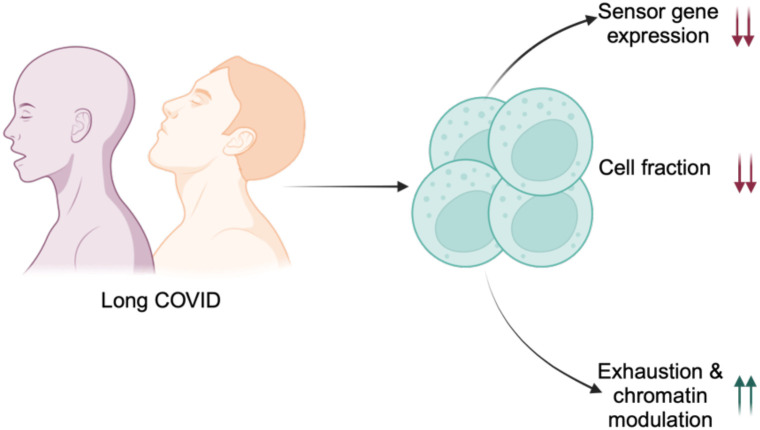

## Highlights

- LTCS patients retain high SARS-CoV-2 antibodies but show suppressed cytokines and loss of NK-cell abundance and function.
- Single-cell RNA-seq reveals NK-cell exhaustion and impaired neuronal/sensory pathways and links immune dysfunction to hallmark symptoms such as fatigue, loss of smell, and cognitive impairment.

## Introduction

Against the backdrop of more than 797 million confirmed severe acute respiratory syndrome coronavirus 2 (SARS-CoV-2) infections worldwide, an estimated 10%–40% of individuals experience prolonged symptoms lasting ≥12 weeks after the acute phase of coronavirus disease 2019 (COVID-19), collectively referred to as long-term COVID-19 syndrome (LTCS) or “long COVID” (1–4). In the literature, long COVID is sometimes defined as symptoms lasting 4–12 weeks (1–3 months), whereas *post-COVID* refers to symptoms persisting beyond 3 months (5). To avoid ambiguity, we adopted the term “LTCS” or “long COVID” throughout this manuscript for all patients (≥12 weeks or 3 months—median time). The World Health Organization (WHO) has formally standardized the definition of LTCS within the International Classification of Diseases (ICD-10) framework (6). Recognizing LTCS conditions as a distinct clinical entity marked an essential step toward improving patient care and resource allocation. LTCS represents a heterogeneous and often debilitating syndrome, encompassing a spectrum of persistent symptoms that range from fatigue and myalgia to respiratory impairment and neurocognitive dysfunction (7–9).

Conservative estimates indicate that approximately one in eight individuals worldwide are currently living with LTCS, underscoring a major and enduring global health and socioeconomic burden (10–13). LTCS affects all genders and age groups, though prevalence and severity vary by demographic: individuals aged 30–50 years show the highest rates of diagnosis, and women appear disproportionately affected (14, 15). Over 200 symptoms have been documented, reflecting multisystem involvement encompassing cardiovascular, thrombotic, cerebrovascular, and metabolic complications (including type 2 diabetes), as well as chronic fatigue syndromes such as myalgic encephalomyelitis/chronic fatigue syndrome (ME/CFS), dysautonomia, cognitive dysfunction (“brain fog”), and postural orthostatic tachycardia syndrome (POTS) (2, 16). Recent studies point to a complex and multifactorial pathophysiology, implicating diverse mechanisms such as serotonin depletion contributing to cognitive impairment (17), skeletal myopathy linked to post-exertional malaise (18), and persistent platelet activation driving thromboinflammation (19). The broad heterogeneity in age, sex, and symptom profiles likely reflects differences in clinical definitions, study methodologies, and acute disease severity (20). Despite its high global incidence, no definitive diagnostic tests, targeted therapies, or validated clinical biomarkers currently exist, further compounding diagnostic uncertainty and patient stigmatization (21).

Given the wide spectrum of LTCS symptoms, the molecular mechanisms driving the transition from acute infection to chronic illness remain poorly understood (4). Several immune-centered hypotheses have been proposed, encompassing viral persistence, reactivation of latent non-SARS-CoV-2 viruses, residual viral antigens in tissues, microbial dysbiosis, chronic tissue injury, autoantibody production, and sustained inflammation (2, 22–24). Persistent SARS-CoV-2 RNA and protein have been detected in multiple organs months after infection, with particularly high abundance in immune cells—especially monocytes (25–27). Such viral persistence is known to induce long-term immune dysregulation, a phenomenon previously described in chronic infections caused by Epstein–Barr virus (EBV) and human herpesvirus 6 (HHV-6) (28, 29). Beyond viral factors, host and societal determinants—including female gender, age, pre-existing comorbidities, smoking, and socioeconomic or ethnic disparities—also contribute to increased LTCS susceptibility (15, 30). Collectively, these findings suggest that LTCS arises from the interplay of persistent viral antigens, immune dysregulation, and host–environmental factors, together driving the path to chronic disease.

Anomalous cellular and humoral immune responses are increasingly recognized as central to the pathogenesis of LTCS (29, 31, 32). One of the first comprehensive immunophenotyping studies demonstrated that LTCS patients exhibit sustained activation of innate immune cells, reduced frequencies of naive T and B cells, and persistently elevated type I (IFN-β) and type III (IFN-λ1) interferons—findings that remained evident even 8 months post-infection compared with healthy controls (31). Concurrently, levels of CXCL10, soluble TIM-3 (sTIM-3), and IL-8 declined over time in both LTCS and convalescent (CONV) cohorts, suggesting ongoing but dysregulated immune resolution (31). Non-lymphoid immune populations in LTCS display a distinct HLA-DR^+^CD38^+^ activation phenotype, while the absence of inactivated naive T and B cells further points to chronic immune perturbation (31, 33). In parallel, mast-cell activation has been implicated in mediating histamine-related and dysautonomic symptoms (34). Beyond systemic inflammation, virus-induced neuroinflammatory changes have been proposed to underlie the relapsing neurological and cognitive manifestations of LTCS (35, 36). Persistent vascular inflammation and complement dysregulation also appear to contribute to thromboinflammatory end-organ sequelae (19, 37). Taken together, current evidence underscores heterogeneous yet interconnected immune alterations, but no unified model has yet explained how immune dysregulation determines disease persistence or recovery trajectories (e.g., 6 months *vs*. >2 years). Consequently, multiple mechanistic pathways remain open for discovery, particularly those that could yield predictive biomarkers or therapeutic targets.

Most previous investigations into LTCS have analyzed plasma or peripheral blood mononuclear cells (PBMCs) using immunoassay and flow cytometry-based approaches (21, 38–40). More recently, bulk and single-cell RNA sequencing (scRNA-seq) have been applied to delineate the transcriptomic landscape of LTCS (21, 41). Notably, a longitudinal study of 165 acutely infected, hospitalized individuals profiled whole blood transcriptomes from the acute to post-acute phases (42). The authors identified two distinct gene-expression trajectories emerging early during infection, critically shaped by the innate and adaptive immune responses. One trajectory featured high expression of immunoglobulin-related genes associated with anti-spike antibody titers, whereas the other showed lower immunoglobulin gene expression, indicative of suboptimal or non-specific antibody production in individuals predisposed to post-acute sequelae (42). These findings underscore the molecular heterogeneity of LTCS patients defined by immune gene signatures. In line with this, recent scRNA-seq studies have revealed myeloid cell dysregulation (25) and T-cell functional exhaustion, persistent inflammation, and a blunted adaptive immune response in LTCS (41, 43). However, the molecular crosstalk between adaptive T cells and innate effectors—particularly natural killer (NK) cells—remains poorly understood. Addressing this gap is critical to elucidate the immune networks underlying chronic LTCS pathology.

Here, we provide an integrated characterization of the humoral and cellular immune landscape in LTCS patients (*n* = 114) using multiplex cytokine and antibody assays, multiparametric flow cytometry, and scRNA-seq. We found that LTCS individuals retained elevated SARS-CoV-2-specific IgG titers—targeting the receptor-binding domain (RBD1), spike S1, and nucleocapsid (N) antigens—up to 500 days post-infection, relative to healthy controls (HC). To dissect how circulating inflammatory mediators influence immune-cell function and contribute to sustained immune dysregulation, we analyzed cytokine profiles alongside cellular phenotypes. LTCS patients exhibited suppressed levels of both pro- and anti-inflammatory cytokines compared with CONV or uninfected HC cohorts. Collectively, our multi-omic data provide a comprehensive cellular and molecular map of LTCS, revealing a striking quantitative and functional loss of NK cells as a potential central driver of LTCS pathogenesis. These findings position NK-cell dysfunction as a key mechanistic hallmark of this enigmatic post-viral syndrome.

## Materials and methods

### Patient cohort recruitment, ethics statement, and patient samples used for different methods

The patient materials analyzed in this study were obtained from the registered clinical trial “COVID-19 Next-Generation Sequencing (COVID-NGS)” (ClinicalTrials.gov Identifier: NCT04364828). The details of the trial can be accessed at: https://clinicaltrials.gov/ct2/show/NCT04364828. The study follows a hybrid design approved by the Ethics Committee of the University of Tübingen (approval no. 286/2020B1), integrating both retrospective and prospective components. The retrospective arm includes individuals who had recovered from SARS-CoV-2 infection and were evaluated at the Tübingen University Hospital Post-COVID Care Unit between June 2020 and December 2021. The prospective arm comprises ongoing sample collection and longitudinal multi-omics analyses conducted within the COVID-NGS cohort, aimed at elucidating the long-term immunological and molecular sequelae of SARS-CoV-2 infection. This study was performed in accordance with the Declaration of Helsinki, and all patients provided written consent.

### Inclusion criteria

Participants were enrolled as healthy controls, convalescent COVID-19 patients, or individuals with long-term COVID-19 syndrome. Healthy controls were negative for SARS-CoV-2-specific IgG antibodies against spike S1, receptor-binding domain (RBD), and nucleocapsid (N), as well as IgM. CONV and LTCS participants were positive for SARS-CoV-2-specific IgG and/or IgM antibodies. All participants had plasma samples available for downstream immunological analyses.

### Exclusion criteria

Plasma samples exhibiting red blood cell lysis were excluded from the 3-plex antibody assay and 13-plex cytokine panel. Samples with antibody below the assay detection limits were excluded from the final analysis. For the 13-plex cytokine assay, values below the lower limit of detection were retained for all samples, except for those in which no cytokine signal was detected. Samples were excluded from 14-color flow cytometry and scRNA-seq analyses if cells (<1.0 × 10^6^) were insufficient, cell viability (<70%–80%) was inadequate, or predefined quality control criteria were not met. Consequently, not all enrolled participants were included in each assay; the number of samples analyzed per experimental platform is reported in **Supplementary Tables 1**-**4**.

Some plasma samples from the HC, CONV, and LTCS cohorts were previously analyzed for cytokine levels using the 13-plex Inflammatory Panel 1 in our earlier studies (44).

### Patient demographics and cohort description

In total, we analyzed samples from 215 individuals, comprising three groups: uninfected healthy controls (HC; *N* = 66), convalescent (CONV; *N* = 35), and LTCS (*N* = 114) patients. The classification of LTCS cases followed the National Institute for Health and Care Excellence (NICE, UK) guideline issued in November 2020, which was in effect during participant recruitment (3). The median time from acute infection to sample collection was 152 days for LTCS patients and 128 days for CONV individuals, both meeting the duration criteria outlined by the NICE and World Health Organization (WHO) definitions for post-COVID-19 condition. HC were seronegative for SARS-CoV-2 IgG and IgM, whereas CONV and most LTCS individuals were seropositive; however, a subset of LTCS patients had undetectable antibody levels, consistent with heterogeneous immune responses observed in long COVID. To account for differences in sampling time between CONV and LTCS patients, we conducted sensitivity analyses restricted to samples collected within ≤200 days post-infection and repeated the primary antibody comparisons. We also fitted linear models (primary regression model—time-adjusted comparison and reduced model if interaction was not significant) with antibody level as the dependent variable and included days post-infection and cohort (LTCS *vs*. CONV) as predictors, with an additional cohort × time interaction term to test whether the time-dependent antibody trajectory differed by groups (**Supplementary Table 5**).

### PBMCs and plasma isolation from an EDTA blood sample

Peripheral blood (2–6 mL) was collected in EDTA tubes and processed within 2 h of collection. For plasma isolation, 0.5–1.0 mL of blood was transferred into a microcentrifuge tube and centrifuged at 2,000×*g* for 10 min at room temperature (RT). The supernatant plasma layer (250–500 µL) was carefully aspirated, transferred into pre-labeled cryovials, and stored at –80°C until use.

For PBMC isolation, blood was diluted 1:1 with phosphate-buffered saline (DPBS; #D8537-500ML, Sigma-Aldrich, Germany) and layered gently over Pancoll Human (#P04-601000, PAN-Biotech, Germany; 15 mL per 50 mL tube) to form two distinct phases. Samples were centrifuged at 400×*g* for 22 min at RT without brake. The buffy coat interface (PBMC layer) was collected using a sterile Pasteur pipette, transferred to a fresh 50-mL tube containing 20 mL of PBS, and centrifuged again at 400×*g* for 5 min at RT. The cell pellet was resuspended in 2–3 mL of PBS and transferred to a 15-mL tube for counting using Trypan Blue dye exclusion on an automated cell counter (Bio-Rad, Germany). PBMCs were cryopreserved in freezing medium containing a final concentration of 15% DMSO, 35% FBS, and 50% DPBS and stored at −80°C until further use.

### 3-Plex SARS-CoV-2 serological IgG panel antibody assay

To quantify SARS-CoV-2 serological IgG antibodies using flow cytometry, plasma samples were analyzed for three key viral proteins—S1, RBD, and N—using the LEGENDplex™ SARS-CoV-2 Serological IgG Panel (3-plex), a bead-based multiplex assay (#741132; BioLegend, USA). Frozen plasma samples were thawed at RT, arranged in chronological order, and recorded with corresponding serial numbers, sample IDs, and PBMC isolation dates. For dilution, 198 µL of assay buffer (from the kit) was added to a labeled Eppendorf tube, followed by 2 µL of plasma, and vortexed for 3–5 s. The lyophilized SARS-CoV-2 serological standard was reconstituted with 250 µL of 1× assay buffer, mixed thoroughly, and left at RT for 15 min. The reconstituted solution was designated as the top standard (C7). Six additional tubes were labelled C6–C1 to prepare 1:4 serial dilutions of the top standard using 75 µL of 1× assay buffer per tube. The assay buffer alone (C0) served as the negative control. For standard curve preparation, 25 µL of 1× assay buffer was added to the first two columns of the plate, and 25 µL of each standard (C7–C0) was added in duplicate. For the samples, 46.5 µL of assay buffer and 3.5 µL of diluted plasma (final 1:800 dilution) were added per well in a 96-well plate. Subsequently, 25 µL of 3-plex beads were vortexed briefly (10–15 s) and added to each well. The plate was sealed, covered with aluminum foil, and incubated for 2 h at 800 rpm on a shaker at RT. After incubation, the plate was centrifuged at 250×*g* for 5 min, and the supernatant was discarded by inverting the plate. The wells were washed with 200 µL of 1× wash buffer and centrifuged again at 250×*g* for 5 min, and the supernatant was removed. Next, 25 µL of detection antibody was added to each well, followed by incubation for 1 h at 800 rpm. Then, 25 µL of streptavidin–phycoerythrin (SA–PE) was added, and the plate was incubated for an additional 30 min under the same conditions. After incubation, the wells were washed again with 200 µL of 1× wash buffer and centrifuged, and the supernatant discarded. Finally, 150 µL of 1× wash buffer was added to each well, and the plate was stored at 4°C until acquisition. Samples were acquired on a flow cytometer, and antibody concentrations were quantified using the BioLegend LEGENDplex™ Data Analysis Software, expressed in µg/mL.

### 13-plex COVID cytokine storm panel 1 assay

The COVID-19 Cytokine Storm Panel 1 (13-plex) (BioLegend, USA) is a multiplex, fluorescence-encoded, bead-based assay designed to quantify multiple cytokines and chemokines simultaneously by flow cytometry. The panel allows concurrent measurement of 13 human cytokines, namely, IL-6, MCP-1 (CCL2), G-CSF, IFN-α2, IL-2, IFN-γ, IL-7, IL-1RA, IL-8 (CXCL8), TNF-α, IP-10 (CXCL10), MIP-1α (CCL3), and IL-10. Frozen plasma samples were thawed at RT. For each sample, 15 µL of assay buffer and 15 µL of plasma were combined in a labeled microcentrifuge tube to prepare a 2× diluted analyte. The lyophilized 13-plex cytokine standard was reconstituted in 250 µL of 1× assay buffer, mixed thoroughly, and incubated at RT for 15 min to form the top standard (C7). A 1:4 serial dilution was then prepared to generate six additional standards (C6–C1) using 75 µL of 1× assay buffer in each tube; the assay buffer alone (C0) served as the blank control. For the standard curve, 25 µL of 1× assay buffer was added to the first two columns of the 96-well assay plate, followed by 25 µL of each standard (C7–C0) in duplicate. For sample wells, 25 µL of assay buffer and 25 µL of diluted plasma (final 1:2 dilution) were added per well. Subsequently, 25 µL of 13-plex bead mixture was vortexed briefly (10–15 s) and added to each well. The plate was sealed, protected from light with aluminum foil, and incubated for 2 h at 800 rpm, RT on a shaker. After incubation, the plate was centrifuged at 250×*g* for 5 min, the supernatant was discarded, and wells were washed with 200 µL of 1× wash buffer using a multichannel pipette. Following a second centrifugation under identical conditions, 25 µL of detection antibody was added to each well, and the plate was incubated for 1 h at 800 rpm, RT. Subsequently, 25 µL of SA–PE was added and incubated for an additional 30 min. The wells were washed again, centrifuged, resuspended in 150 µL of 1× wash buffer, and then stored at 4°C until acquisition. Samples were acquired on a flow cytometer, and data analyzed using the BioLegend LEGENDplex™ Data Analysis Software. Cytokine and chemokine concentrations were calculated from the standard curves and expressed in pg/mL.

### Surface and intracellular staining of PBMC for immunophenotyping

Samples were thawed for exactly 2 min at 37°C in a water bath. The thawed cell suspension was transferred into a 50-mL Falcon tube. To the cryovial, 1 mL of pre-warmed RPMI 1640 medium supplemented with 10% fetal bovine serum (FBS) and 1% antibiotic–antimycotic (all from Thermo Fisher Scientific, Germany; referred to as *complete RPMI medium*) was added and mixed thoroughly. The complete medium was then added dropwise to the Falcon tube containing the cells. This step was repeated with successive additions of 2, 4, 8, and 16 mL of complete medium, each added dropwise to allow gradual dilution and minimize osmotic shock. The tube was gently inverted 2–3 times to mix and centrifuged at 400×*g* for 5 min at RT. After centrifugation, the supernatant discarded, leaving approximately 1 mL of medium above the pellet. The cells were then washed again with 20 mL of complete medium and centrifuged under the same conditions, and the supernatant was discarded. PBMCs were resuspended, counted, and assessed for viability. Approximately 0.5–1.0 × 10^6^ cells were transferred into each well of a 96-well plate and centrifuged at 600×*g* for 5 min at RT. The supernatant was removed by gently flicking the plate, and 17.5 µL of PBS was added to each well.

For surface staining (14-color staining—13 surface antibodies and 1 intracellular antibody), an antibody cocktail (13 antibodies) was prepared per well consisting of 5 µL staining buffer, 2 µL of each of 12 antibodies (anti-CD3, anti-CD4, anti-CD8a, anti-CD-19, anti-CD45RA, anti-HLA-DR, anti-CD38, anti-CD154, anti-CCR7, anti-CD14, anti-CD16, and anti-CD69; total = 24 µL), and 3.5 µL of anti-CD56 antibody, yielding a final volume of 32.5 µL per well (**Table 1**). The antibody cocktail was added to each well, the plate was covered with aluminum foil, and incubated for 40–45 min at RT. After incubation, 150 µL of PBS was added, and the plate was centrifuged at 600×*g* for 5 min at RT. The supernatant was discarded by flicking the plate.

**Table 1:** Key resource table for reagents, tools, and codes used for the study.

Reagent or resource	Source	Identifier
Antibodies		
Anti-human CD3 eFlour™ 450 (clone UCHT1)	Thermo Fisher Scientific (eBioscience™)	Cat#48-0038-42
Anti-human CD4 Super Bright™ 600 (clone SK-3)	Thermo Fisher Scientific (eBioscience™)	Cat#63-0047-42
Anti-human CD8a PerCP-eFlour™ 710 (clone SK1)	Thermo Fisher Scientific (eBioscience™)	Cat#46-0087-42
Anti-human CD19 eFlour™ 506 (clone HIB19)	Thermo Fisher Scientific (eBioscience™)	Cat#69-0199-42
Anti-human CD56 PE (clone MEM-188)	Thermo Fisher Scientific (eBioscience™)	Cat#MA1-19638
Anti-human CD45RA PE-Cyanine7 (clone HI100)	Thermo Fisher Scientific (eBioscience™)	Cat#25-0458-42
Anti-human HLA-DR Alexa Fluor™ 647 (clone L243)	Thermo Fisher Scientific (Invitrogen)	Cat#A51010
Anti-human CD38 PE-eFlour™ 610 (clone HIT2)	Thermo Fisher Scientific (eBioscience™)	Cat#61-0389-42
Anti-human CD154 (CD40 Ligand) PE-Cyanine5 (clone 24-31)	Thermo Fisher Scientific (eBioscience™)	Cat#15-1548-42
Anti-human CD197 (CCR7) Brilliant Violet 785™ (clone G043H7)	BioLegend	Cat#353230
Anti-human CD14 Alexa Fluor™ 700 (clone 61D3)	Thermo Fisher Scientific (eBioscience™)	Cat#56-0149-42
Anti-human CD16 Super Bright™ 702 (clone 3G8)	Thermo Fisher Scientific (eBioscience™)	Cat#67-0166-42
Anti-human CD69 APC-eFluor™ 780	Thermo Fisher Scientific (eBioscience™)	Cat#47-0699-42
Anti-human FOXP3 FITC (clone PCH101)	Thermo Fisher Scientific (eBioscience™)	Cat#11-4776-42
Anti-human Granzyme B Alexa Fluor™ 488 (clone 351927)	Thermo Fisher Scientific	Cat#MA5-23639
Anti-human Perforin PE-Cyanine7 (clone delta G9 or dG9)	Thermo Fisher Scientific (eBioscience™)	Cat#25-9994-42
Anti-human Granzyme A PE (clone CB9)	Thermo Fisher Scientific (eBioscience™)	Cat#12-9177-42
Anti-human TNF-α eFluor™ 450 (clone MAb11)	Thermo Fisher Scientific (eBioscience™)	Cat# 48-7349-42
eBioscience™ Foxp3/Transcription Factor Staining Buffer Set	Thermo Fisher Scientific (eBioscience™)	Cat#00-5523-00
Chemicals and other reagents		
RPMI 1640	Thermo Fisher	Cat# 61870036
Antibiotics/antimycotics	Thermo Fisher	Cat# 15240062
FBS	Thermo Fisher	Cat# A5256701
Ficoll Hypaque	PAN-Biotech	Cat# P04-601000
LEGENDplex™ COVID-19 Cytokine Storm Panel 1 (13-plex) w/ VbP	BioLegend	Cat#741091
LEGENDplex™ SARS-CoV-2 Serological IgG Panel (3-plex) w/VbP	BioLegend	Cat#741132
Cell Staining Buffer	BioLegend	Cat#420201
N 184 DeNovix Acridine Orange/Propidium Iodide Assay	DeNovix	Cat#TN184
Trypan Blue	Sigma-Aldrich	Cat#T8154-100ML
High Sensitivity DNA kit	Thermo Fisher Scientific	Cat# Q33230
SP flow cell (200 cycles)	Illumina, USA	Cat# 20040719
Super Bright Complete Staining Buffer	Thermo Fisher Scientific (eBioscience™)	Cat#SB-4401-42
UltraComp eBeads™ Compensation Beads	Thermo Fisher Scientific (Invitrogen)	Cat# 01-2222-42
ECW02130 Evercode™ WT v2	Parse Biosciences	Cat#ECW02130
Software		
Inkscape v1.2.2	Inkscape	https://inkscape.org/release/inkscape-1.2/
R version V4.4.0		https://cran.r-project.org/bin/windows/base/old/
R Studio Version 2024.09.1 + 394 (2024.09.1 + 394)		https://posit.co/download/rstudio-desktop/
Microsoft^®^ Excel for Mac Version 16.96 (25041326)		Microsoft 365 Subscription
fgsea v1.30.0		https://bioconductor.org/packages/release/bioc/html/fgsea.html
clusterProfiler v4.12.6		https://bioconductor.org/packages/release/bioc/html/clusterProfiler.html
EnhanceVolcano v1.22.0		https://bioconductor.org/packages/release/bioc/html/EnhancedVolcano.html
FlowJo 10.10	FlowJo	https://www.flowjo.com/flowjo/download
CellRanger software v3.0.1	10x Genomics	https://github.com/10XGenomics/cellranger
Human reference genome GRCh38 v3.0.0		https://www.ncbi.nlm.nih.gov/datasets/genome/GCF_000001405.26/
Seurat 5.2.0		https://satijalab.org/seurat/
ggplot2 3.5.3		https://ggplot2.tidyverse.org
Tidyverse v2.0.0		https://www.tidyverse.org
gridExtra v2.3		https://cran.r-project.org/web/packages/gridExtra/index.html
SeuratWrappers v0.3.5		https://github.com/satijalab/seurat-wrappers
Presto		https://github.com/immunogenomics/presto
Dplyr		https://dplyr.tidyverse.org
patchwork		https://cran.r-project.org/web/packages/patchwork/index.html
cowplot v		https://cran.r-project.org/web/packages/cowplot/vignettes/introduction.html
SeuratData v0.2.2.9001		https://github.com/satijalab/seurat-data
scales v1.3.0		https://scales.r-lib.org
reshape2 v1.4.4		https://cran.r-project.org/web/packages/reshape2/index.html
scattermore v1.2		https://cran.r-project.org/web/packages/scattermore/index.html
hdf5r v1.3.12		https://cran.r-project.org/web/packages/hdf5r/index.html
ComplexHeatmap v2.20.0		https://bioconductor.org/packages/release/bioc/html/ComplexHeatmap.html
dittoseq v1.16.0		https://bioconductor.org/packages/release/bioc/html/dittoSeq.html
viridis v0.6.5		https://cran.r-project.org/web/packages/viridis/vignettes/intro-to-viridis.html
pathview v1.44.0		https://www.bioconductor.org/packages/release/bioc/html/pathview.html
enrichplot v1.24.4		https://bioconductor.org/packages/release/bioc/html/enrichplot.html
wordcloud v2.6		https://cran.r-project.org/web/packages/wordcloud/index.html
msigdbr v10.0.1		https://cran.r-project.org/web/packages/msigdbr/index.html
DOSE v3.30.5		https://www.bioconductor.org/packages/devel/bioc/vignettes/DOSE/inst/doc/DOSE.html
Parallel v4.4.2		https://cran.r-project.org
ggpubr v0.6.0		https://cran.r-project.org/web/packages/ggpubr/index.html
FSA v0.9.6		https://cran.r-project.org/web/packages/FSA/index.html
Rstatix v0.7.2		https://cran.r-project.org/web/packages/rstatix/index.html
Deposited data		
scRNA-seq data	This study	Zenodo accession ID: https://zenodo.org/uploads/14886569 and sequencing data available through GEO accession; GSE320507.
Code used for single-cell RNA sequencing analysis and figure generation	This study	https://github.com/ysinghbt/scLTCS

For intracellular staining, 100 µL of fixation buffer (Thermo Fisher Scientific, formerly eBioscience) was added to each well, and the plate was incubated for 45 min at RT. After incubation, the wells were washed with 100 µL of 1× permeabilization buffer (Thermo Fisher Scientific, formerly eBioscience), and the supernatant was discarded. Then, 38 µL of 1× permeabilization buffer was added to each well. An intracellular antibody cocktail was prepared by mixing 10 µL of 1× permeabilization buffer with 2 µL of FoxP3–FITC antibody per sample, and 12 µL of this mixture was added to each well. The plate was covered and incubated for 45 min at RT. After incubation, 150 µL of PBS was added, and the wells were washed once more. The supernatant was discarded, and the wells were resuspended in 150 µL of PBS. The plate was kept at 4°C until data acquisition on a BD Fortessa flow cytometer (4-laser configuration). For intracellular cytokine and effector protein staining (TNF-α, perforin, and granzyme), separate staining experiments were performed on independent PBMC samples following similar procedures with minor modifications, as described previously (45).

### SPLiT-seq scRNA-seq using Parse Biosciences

#### PBMC fixation for SPLiT-seq

Cryopreserved PBMCs were thawed in a 37°C water bath until only a small amount of frozen material remained, as described in the surface and intracellular staining section. PBMCs (0.5 × 10^6^–2 × 10^6^ cells) were processed using the Evercode Fixation Kit v2.02 (Parse Biosciences, Seattle, WA, USA; SKU: ECF2001) according to the manufacturer’s protocol.

Briefly, PBMCs were counted using a TC20™ Automated Cell Counter (Bio-Rad), and 0.5 × 10^6^–2 × 10^6^ live cells per sample were transferred into a 15-mL tube and kept on ice. The cells were centrifuged at 400×*g* for 5 min, the supernatant was discarded, and the pellet was resuspended in 750 µL of cold Cell Prefixation Buffer. The suspension was passed through a 40-µm cell strainer into a new 15 mL Falcon tube and kept on ice. Next, 250 µL of Cell Fixation Solution was added to the cell suspension, mixed gently by pipetting up and down three times, and incubated on ice for 10 min. Following fixation, 80 µL of Cell Permeabilization Solution was added, mixed with pipetting three times, and incubated on ice for an additional 3 min. After incubation, 1 mL of Cell Neutralization Buffer was added, the tube was gently inverted once to mix, and cells were centrifuged at 400×*g* for 5 min. The supernatant was discarded, and the pellet was resuspended in 100 µL of cold cell buffer. Subsequently, 5 µL of DMSO was added, the mixture was pipetted gently three times to avoid bubble formation, and the cells were incubated on ice for 1 min. Cells were then counted and divided equally into two cryovials, which were placed in a freezing container under standard freezing conditions for fixed cells and stored at −80°C until library preparation. In total, approximately 700,000 cells from 48 donors were processed, yielding an average of 2,000–2,500 cells per donor.

### Barcoding and library preparation

Prior to cell barcoding and library preparation, fixed PBMC samples were removed from −80°C, thawed in a 37°C water bath, placed on ice, and recounted for viability and cell number. Libraries were constructed using the Single Cell Whole Transcriptome Kit v2 (Parse Biosciences, Seattle, WA, USA) according to the manufacturer’s instructions. The resulting scRNA-seq libraries were sequenced on an Illumina NovaSeq 6000 platform, generating 200 bp paired-end reads with an average depth of >20,000 reads per cell.

### Raw data processing and quality control

Raw sequencing reads were processed and aligned to the human reference genome GRCh38 (hg38) using the Parse Biosciences split-pipe v1.1.2 pipeline to generate cell-by-gene count matrices for each sample. The matrices were imported into Seurat v5.1.0 for downstream analysis. Cells were filtered out if they contained <800 or >10,000 unique molecular identifiers (UMIs), <200 or >5,000 detected genes, or ≥10% mitochondrial gene content. Potential doublets were identified and removed using DoubletFinder v1.18.0. Gene counts were log-normalized, and the top 2,000 highly variable genes were selected for principal component analysis (PCA).

To correct for batch effects, datasets from the three groups—HC, CONV, and LTCS—were integrated using canonical correlation analysis (CCA). Dimensionality reduction was performed with uniform manifold approximation and projection (UMAP) based on the first 20 principal components. Unsupervised clustering was conducted using a graph-based algorithm with a resolution of 1.0, and cell types were annotated according to canonical marker genes. Doublet detection was performed using the scDoubletFinder algorithm to identify and remove potential cell doublets from the single-cell RNA-sequencing data. The method estimates the expected doublet rate based on cell loading density and sequencing depth, generates artificial doublets, and assigns each cell a doublet score by comparing its transcriptional profile to simulated doublets and real singlets. Cells classified as high-confidence doublets according to the recommended scDoubletFinder thresholds were excluded from downstream analyses to ensure data quality and accurate cell-type identification.

### Differential gene expression analysis

Bulk and pseudo-bulk differential expression analyses comparing HC *vs*. LTCS and CONV *vs*. LTCS were performed in Seurat. Cell-type-specific expression profiles were aggregated to create pseudo-bulk gene-count matrices. Differentially expressed genes (DEGs) were identified using DESeq2, applying thresholds of |log_2_ fold change| >1 and adjusted *P <*0.05.

### Gene set enrichment analysis—GO and KEGG

Gene set enrichment analysis (GSEA) was performed to identify significantly enriched Kyoto Encyclopedia of Genes and Genomes (KEGG) pathways and Gene Ontology (GO) terms (biological process, molecular function, and cellular component) associated with DEGs. Enrichment analysis was carried out using overrepresentation analysis (ORA) implemented in clusterProfiler v4.12.6, applying Fisher’s exact test with Benjamini–Hochberg correction for multiple testing. Data visualization was performed using the enrichplot v1.24.4 R package.

### Statistics and data visualization

No *a priori* statistical method was used to predetermine sample size. Samples were selected based on availability and successful quality control during flow cytometry and scRNA-seq analyses for the HC, CONV, and LTCS groups. Randomization was not applied, as the study design involved predefined group comparisons (LTCS *vs*. CONV). Data collection and analysis were not blinded, as the experimentalist was aware of sample group identity. All analyses were conducted on Linux servers using R v4.4.2 and RStudio. Figures were generated in Inkscape, and schematic illustrations were created with BioRender. A complete list of all chemicals, reagents, kits, and tools employed in this research is provided in **Table 1**.

### Key resource table

## Results

### LTCS is associated with an activated humoral immune response coupled to a reciprocal reduction of inflammatory mediators

**Figure 1:**
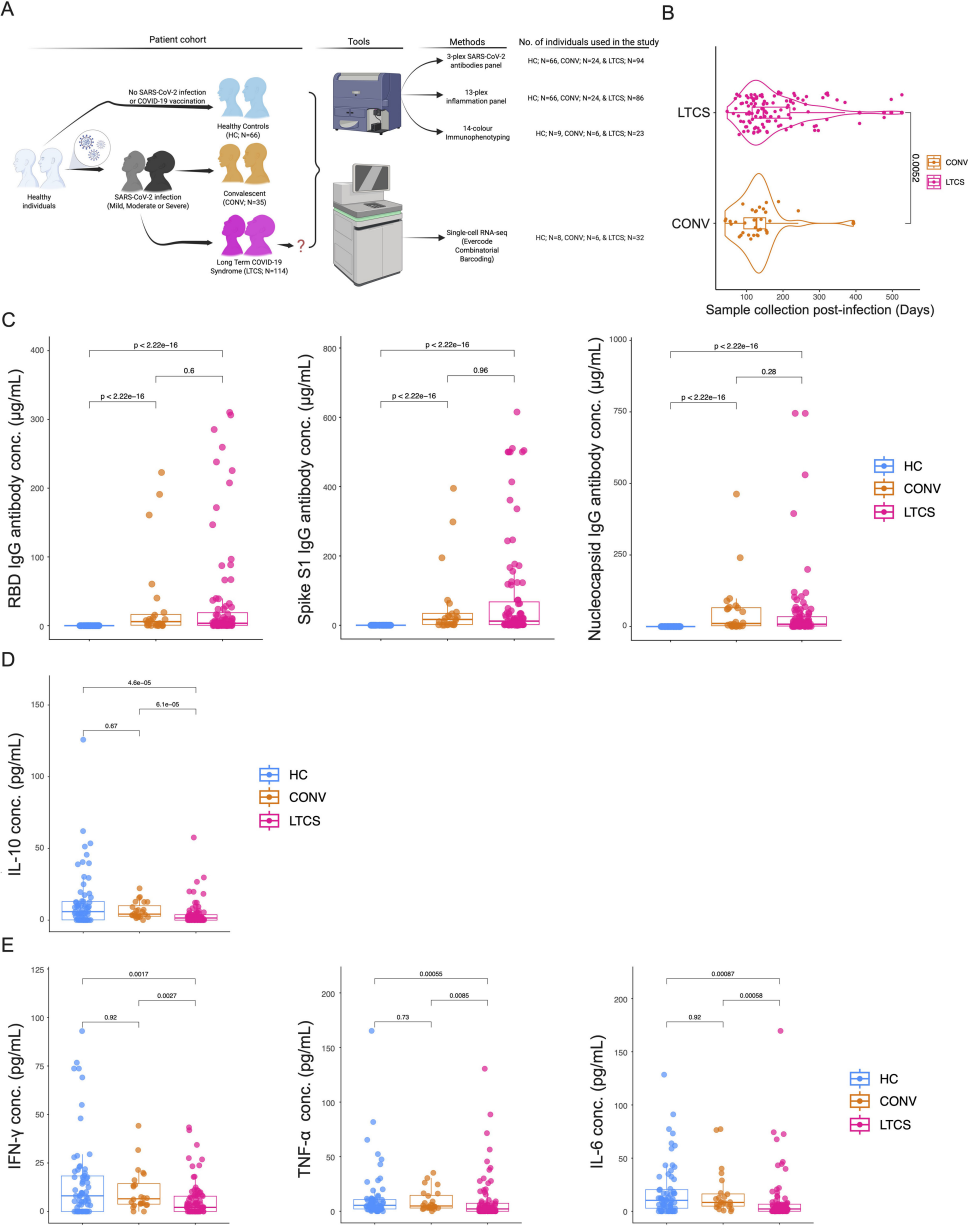
Assessment of IgG antibodies and cytokine levels in long COVID patients. **(A)** Schema showing cohort recruitment strategy: healthy control (HC; blue; *N* = 66), convalescent (CONV; orange; *N* = 35), and long COVID patients (LTCS; pink; *N* = 114). Sample number was denoted as *N* in the main text and the panel figure (made in BioRender). **(B)** Mean sample collection in days for convalescent (*N* = 35) and LTCS (*N* = 114) patients. A *P*-value ≤0.05 is considered statistically significant. **(C)** Increased levels of RBD IgG, spike S1 IgG, and nucleocapsid IgG in LTCS (*N* = 94) compared with HC (*N* = 66). A *P*-value ≤0.05 is considered statistically significant. **(D)** Anti-inflammatory cytokine IL-10 levels were significantly reduced in LTCS (*N*=86) compared with HC (N=66) or CONV (*N*=24). P value ≤ 0.05 is considered significant. **(E)** Pro-inflammatory cytokines IFN-γ, TNF-α, and IL-6 levels were significantly reduced in LTCS (*N*=86) compared with HC (N=66) or CONV (*N*=24). P value ≤ 0.05 is considered significant.

We analyzed samples collected from individuals up to 500 days post-infection during the peak of the pandemic (June 2020–December 2021) who continued to experience post-COVID symptoms. Samples were retrospectively categorized into LTCS and CONV groups based on clinical metadata (**Figure 1A**, **Supplementary Table 1**). The difference in sampling time between the LTCS and CONV cohorts was statistically significant (*P* = 0.005), indicating that CONV participants had recovered earlier, while LTCS patients continued to experience persistent symptoms (**Figure 1B**). Our cohort comprised healthy controls (HC; *N* = 66), CONV individuals (*N* = 35), and LTCS patients (*N* = 114). The average age was 41.9 ± 15.4 years for HC, 62.0 ± 19.2 years for CONV, and 50.2 ± 15.3 years for LTCS participants (**Supplementary Figure 1a**, **Supplementary Table 2**).

Kruskal–Wallis testing revealed that the HC group was significantly younger than both CONV (Padj = 5.2 × 10^-7^) and LTCS (Padj = 2.7 × 10^-3^) individuals. Additionally, CONV participants were significantly older than LTCS patients (Padj = 1.6 × 10^-^³). Age–gender stratification showed that female participants were evenly distributed across groups (**Supplementary Figure 1b**), whereas male CONV participants were significantly older than males in either the HC (Padj = 2.0 × 10^-7^) or LTCS (Padj = 5.2 × 10^-5^) groups. Female participants were predominant in the LTCS cohort (60% women, 40% men; **Supplementary Figure 1c**).

Ten major symptom domains were used to define LTCS patients (12, 44, 46). Among the 114 LTCS patients, over half reported fatigue (54%) and dyspnea (55%), followed by dizziness (18%). Other symptoms included anosmia (16%), ageusia (18%), headache (17%), anxiety (12%), myalgia (12%), and neuropathy (16%), whereas diarrhea and sleep disturbances were less frequent (<4%) (**Supplementary Figure 1d**, **Supplementary Table 1**). These findings are consistent with our previous observations (44). Gender-specific analyses further revealed that concentration deficits, anosmia, and ageusia were 2–3-fold more common in men, while headache occurred 3-fold more frequently in women (**Supplementary Figure 1e**, **Supplementary Table 3**).

Previous studies have reported conflicting findings regarding SARS-CoV-2-specific antibody dynamics in LTCS. One study observed that nucleocapsid (N) and spike S1 IgG antibody levels declined within 6 months following SARS-CoV-2 infection in LTCS patients (13), whereas another reported lower frequencies of detectable neutralizing antibodies and reduced S1/S2 titers in this population (38). In contrast, other studies found no association between N and S IgG antibody levels and LTCS symptoms, as both CONV and LTCS groups exhibited comparable titers (47). Other studies also suggest that antibody patterns may predict LTCS risk, with higher anti-nucleocapsid levels linked to increased risk and higher anti-spike levels associated with reduced risk (48). In our cohort, plasma levels of spike S1, RBD, and N antibodies were measured. All three antibody levels were significantly higher in LTCS (*N* = 94) patients than in HC (*N* = 66) individuals (**Figure 1C**). However, no significant difference was observed between the LTCS (*N* = 94) and CONV (*N* = 24) groups. SARS-CoV-2 infection to sample collection differs between the CONV and LTCS cohorts (**Figure 1B**) and could influence immune readouts, including antibody levels. To evaluate the impact of sampling time, we performed sensitivity analyses restricting the dataset to participants sampled within ≤200 days post-infection, a window in which both groups are represented. Within this restricted subset, we re-ran the antibody comparisons shown in **Figure 1C** and observed results consistent with the full cohort analysis (**Supplementary Figure 2a**). Time post-infection was not significantly associated with antibody levels, and inclusion of this covariate did not materially alter the estimated difference between LTCS and CONV.

During the early pandemic, several studies proposed that chronic hyperinflammation underlies LTCS (31), supported by parallels with other post-viral conditions such as chronic fatigue syndrome (49–51). Conversely, other reports describe LTCS as an immune-exhausted state marked by suppressed cytokine production (39, 52, 53). Based on this, we hypothesized that LTCS patients exhibit a subdued inflammatory response following infection. To test this, we profiled plasma cytokines using a 13-plex cytokine storm panel measuring IL-6, MCP-1 (CCL2), G-CSF, IFN-α2, IL-2, IFN-γ, IL-7, IL-1RA, IL-8 (CXCL8), TNF-α, IP-10 (CXCL10), MIP-1α (CCL3), and IL-10. Consistently, both pro- and anti-inflammatory cytokines were significantly downregulated in LTCS (*N* = 86) compared with the HC (*N* = 66) and CONV (*N* = 24) groups (**Figure 1D**). Notably, the anti-inflammatory cytokine IL-10 was markedly decreased in LTCS compared with both control groups (**Figure 1D**). IFN-γ, TNF-α, IL-6, and IL-8 (CXCL8) were also significantly downregulated relative to the HC or CONV groups (**Figure 1E**, **Supplementary Figure 2b**). IL-1RA was significantly increased in LTCS compared with CONV (**Supplementary Figure 2b**), whereas IL-7 was significantly decreased in LTCS compared with HC, but not with CONV (**Supplementary Figure 2b**). IP-10 (CXCL10) was significantly reduced in LTCS compared with CONV (**Supplementary Figure 2b**). IFN-α2 tended to be lower in LTCS compared with HC, although this difference did not reach statistical significance (**Supplementary Figure 2b**). No significant differences were observed for G-CSF, MCP-1, MIP-1α (CCL3), or IL-2 (**Supplementary Figure 2c**). Collectively, these results demonstrate that LTCS patients exhibit attenuated systemic cytokine profiles, reflecting a dampened pro-inflammatory response despite persistent symptoms, suggestive of immune exhaustion rather than sustained hyperinflammation.

### Individuals with LTCS exhibit altered immune-cell composition and disrupted innate–adaptive interactions

Individuals with LTCS display heterogeneous alterations in immune and inflammatory factors, as reported across studies employing diverse sampling strategies and analytical approaches (25, 33, 54, 55). NK cells are cytotoxic lymphocytes that provide a crucial first line of defense against viral infection and malignancy. While several studies have described abnormal NK-cell frequencies and activation states during acute COVID-19 (56, 57), their role in LTCS remains poorly defined.

To assess the immune landscape, we employed a 14-color flow cytometry panel to characterize major immune-cell subsets—including monocytes, T cells, B cells, and NK cells—from PBMCs derived from HC (*N* = 9), CONV (*N* = 6), and LTCS (*N* = 23) participants. The gating strategy is detailed in **Supplementary Figure 3**.

**Figure 2:**
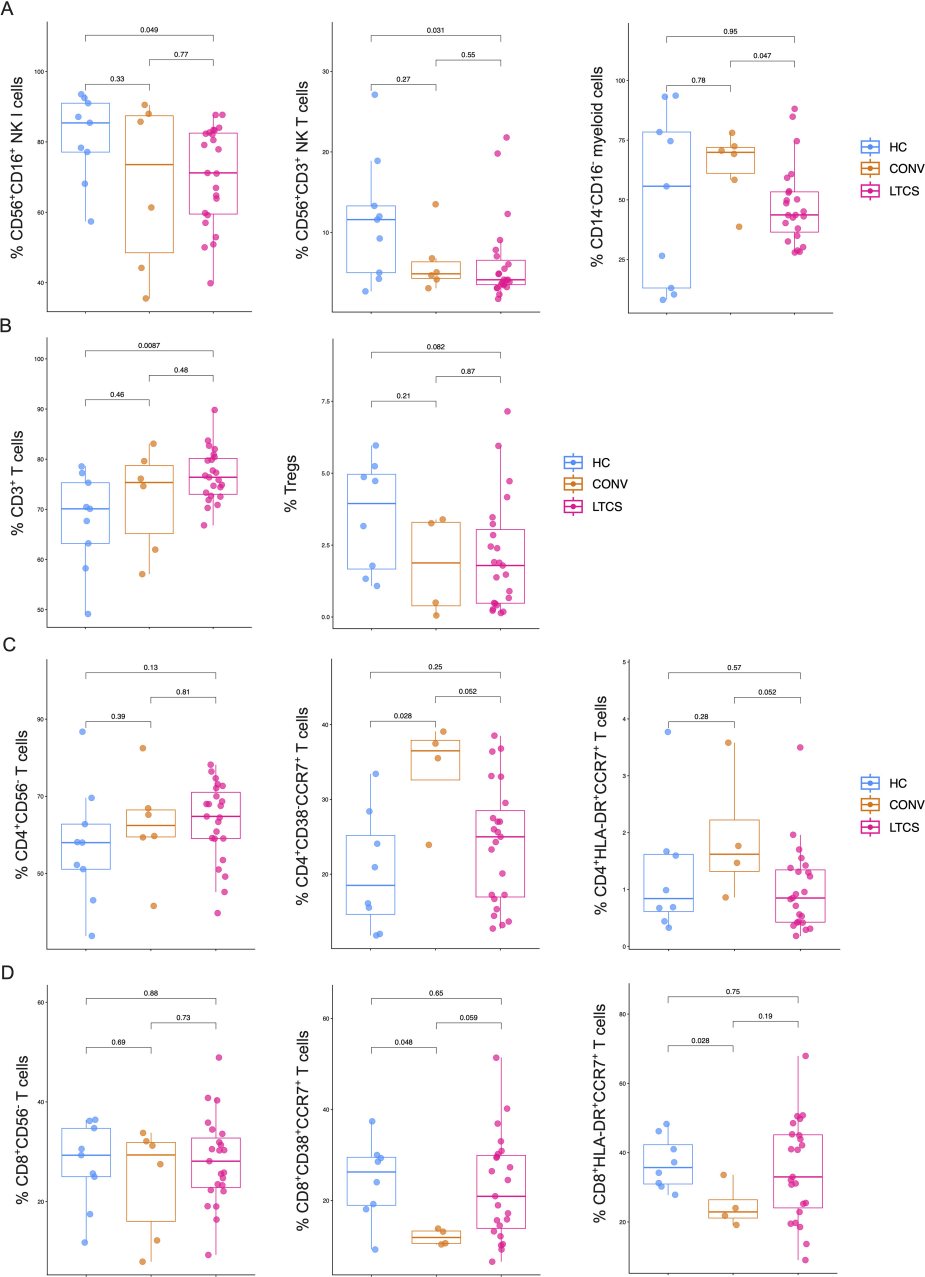
Immunophenotyping of LTCS patients. **(A)** 14-color staining was performed including the basic markers of monocytes and T, B, and NK cells as well as the activation makers HLA-DR, CD38, and CD69. We identified that reduced levels of NK I (left; classical NK cells: CD56^+^CD16^+^ cells) were significantly reduced in the LTCS patient group compared with the control group. Similarly, reduced levels of NKT cells I (middle; classical NK cells: CD56^+^CD3^+^ T cells) were significantly reduced in LTCS patients (*N* = 23) compared with the control (HC) group (*N* = 9). Furthermore, monocytes negative of CD14 and CD16 myeloid cells (right graph) were significantly reduced in the LTCS patient group compared with the convalescent group (*N* = 6). A *P*-value ≤0.05 is considered statistically significant. **(B)** A significant increase in CD3^+^ T cells in the LTCS patient group compared with the control group (HC). A trend toward decreased Tregs in LTCS patients compared with the control group, but not reaching statistical significance. A *P*-value ≤0.05 is considered statistically significant. **(C)** A trend toward increased total CD4^+^ T cells in the LTCS group without reaching statistical significance. Furthermore, a trend toward decreased CD4^+^CD38^−^CCR7^+^ and CD4^+^HLA^−^DR^+^CCR7^+^ T cells in the LTCS patient group compared with the convalescent group, also without statistical significance. However, CD4^+^CD38^−^CCR7^+^ T cells in the convalescent group were statistically significantly higher compared with the control (HC) group. A *P*-value ≤0.05 is considered statistically significant. **(D)** No apparent difference was observed in total CD8^+^ T cells. Overall, CD8^+^CD38^+^CCR7^+^ and CD8^+^HLA-DR^+^CCR7^+^ T cells were statistically significantly reduced in the convalescent group compared with healthy controls. Furthermore, CD8^+^CD38^+^CCR7^+^ and CD8^+^HLA-DR^+^CCR7^+^ T cells tended to be lower in LTCS patients compared with the healthy controls but without statistical significance. A *P*-value ≤0.05 is considered statistically significant.

Our analyses revealed that CD56^+^CD16^+^ NK cells and CD56^+^CD3^+^ NKT cells were significantly reduced in LTCS patients compared with HC (**Figure 2A**), whereas no significant difference was observed between the LTCS and CONV groups. Additionally, CD16^-^CD14^-^ myeloid monocytes were significantly decreased in LTCS compared with CONV individuals (**Figure 2A**). Conversely, the frequency of CD3^+^ T cells was significantly increased in LTCS patients relative to HC (**Figure 2B**). Although regulatory T cells and CD4^+^CD56^-^ conventional T cells were lower in LTCS than in HC, these changes did not reach statistical significance (**Figure 2B**). CD4^+^CD38^+^CCR7^+^ T cells are a specific subset of activated helper CD4^+^ T cells that are a memory-phenotype (expressing CCR7 for lymphoid homing) but also activated/effector-like (expressing CD38), often indicating recent antigen encounter or inflammation, and play roles in antiviral responses, representing a dynamic, activated memory population distinct from naive or fully effector cells (58–60). CD4^+^HLA-DR^+^CCR7^+^ T cells represent a specialized subset of activated central memory T cells that express markers of recent antigen exposure (HLA-DR) while maintaining the ability to home to lymph nodes (CCR7), and these cell subsets are highly functional and recently divided antigen-specific helper cells that are often found in peripheral blood during active infections, including tuberculosis (61). A more granular analysis showed that CD4^+^CD38^+^CCR7^+^ T cells were significantly elevated in CONV compared with HC, while naïve CD4^+^ T cells and CD4^+^HLA-DR^+^CCR7^+^ T cells tended to be reduced in LTCS relative to CONV (**Figure 2C**). Total CD8^+^CD56^-^ T-cell abundance remained unchanged across groups; however, CD8^+^CD38^+^CCR7^+^ and CD8^+^HLA-DR^+^CCR7^+^ T cells were significantly downregulated in CONV compared with HC (**Figure 2D**). In contrast, CD8^+^CD38^+^CCR7^+^ T cells showed a non-significant upward trend in LTCS relative to CONV. Overall, these findings indicate that LTCS is characterized by quantitative and compositional shifts across multiple immune-cell compartments, suggesting that disrupted communication between innate and adaptive immunity may contribute to the persistence of LTCS symptoms.

### scRNA-seq confirms the reduced NK I cell number in LTCS compared with convalescent individuals

LTCS is a heterogeneous condition with diverse clinical manifestations. Although a few studies have performed scRNA-seq analyses in LTCS patients, these were often limited by small cohort sizes, leading to high intragroup variability (25, 33, 41, 55). To investigate the cellular landscape of LTCS at single-cell resolution, we performed scRNA-seq using the Parse Biosciences platform on PBMCs collected from HC (*N* = 8), CONV (*N* = 6), and LTCS (*N* = 32) individuals.

**Figure 3:**
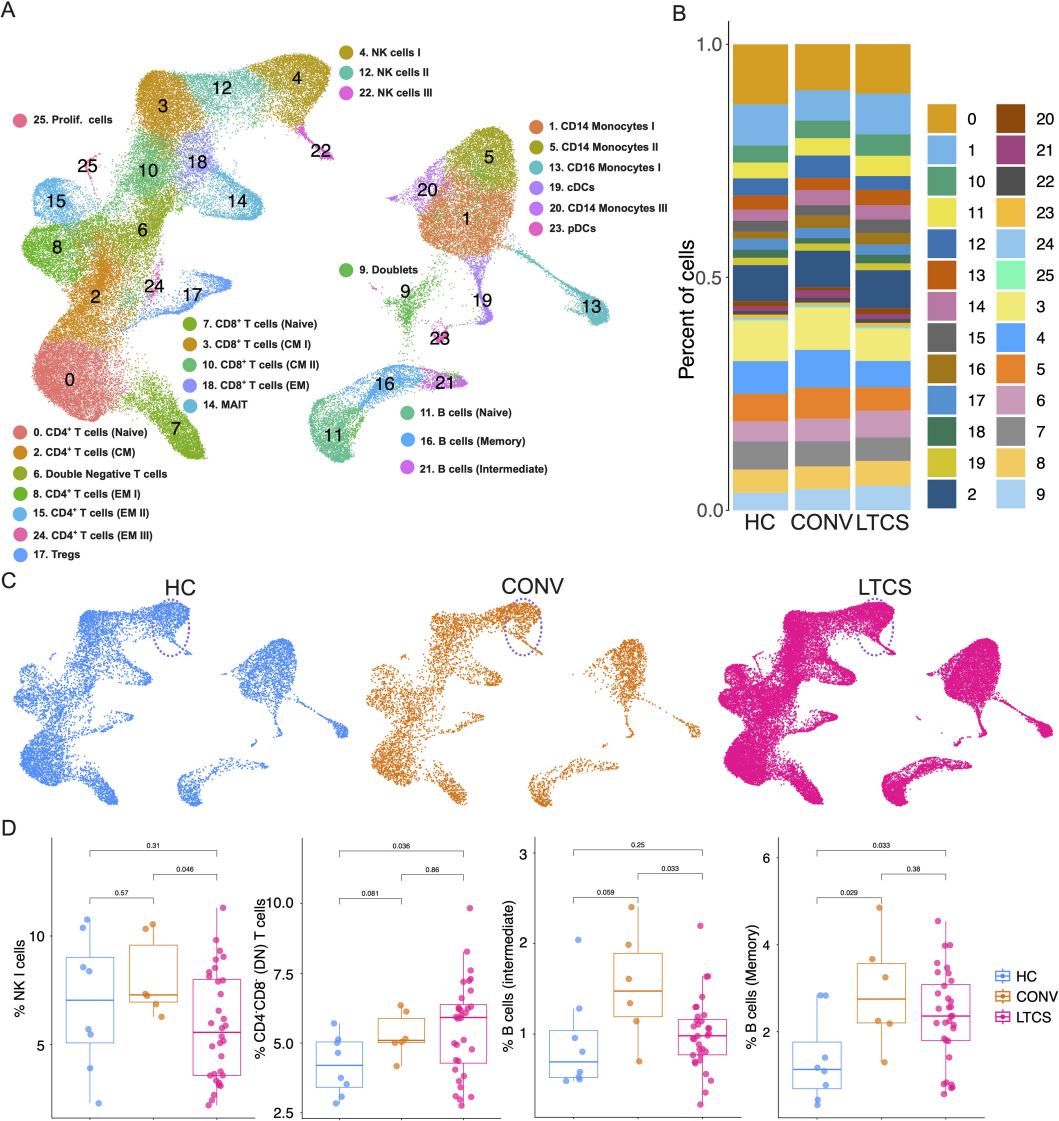
Single-cell RNA profiling identified reduced NK cells in LTCS patients. **(A)** scRNA-seq was performed on the HC (*N* = 8), CONV (*N* = 6), and LTCS (*N* = 32) patient groups. Data analysis was performed based on the Seurat pipeline and identified the cell clusters of different immune cell subsets based on canonical markers in UMAP plots for major cell types. **(B)** Percentage of different immune cell subsets based on cell clusters obtained from UMAP analysis. **(C)** UMAP plots highlighted NK I cells in HC, CONV, and LTCS patients. **(D)** Box and whisker plots show the significantly reduced NK-cell population in LTCS patients compared with the CONV group. Increased (CD4^–^CD8^−^) T cells and B (memory) cells in the LTCS patient group compared with the HC group. B (intermediate) cells tended to be reduced in the LTCS patient group compared with the CONV group. A *P*-value ≤0.05 is considered statistically significant.

The split-and-pool method was used to label PBMCs with unique molecular indices (UMIs), followed by library preparation and sequencing. Raw sequencing data were processed and subjected to rigorous quality control to identify transcriptionally distinct single-cell clusters as described previously (62) (**Supplementary Figures 4a, b**). A total of 70,321 cells passed quality control. Following clustering and UMAP-based dimensionality reduction, 25 major cell clusters were identified (**Figure 3A**). Cross-referencing cluster-specific gene expression profiles with known immune cell marker genes enabled the identification of the principal immune subsets (C0–C25) (62), including monocytes (#C1, 5, 13, 20; ~18.6% of total cells), dendritic cells (#C19, 23; ~2.2%), CD4^+^ T cells (#C0, 2, 8, 15, 24; ~26.6%), CD8^+^ T cells (#C3, 7, 10, 18; ~18.9%), CD3^+^CD4^-^CD8^+^ T cells (#C6; ~4.9%), B cells (#C11, 16, 21; ~6.9%), MAIT cells (#C14; ~2.8%), NK cells (#C4, 12, 22; ~12.1%), and Tregs (#C17; ~2.3%) (**Figure 3B**). Further subclassification of T-cell subsets was performed based on canonical marker expression (**Supplementary Figures 4c, d**).

UMAP visualization revealed distinct distributions of immune cell populations across the LTCS, CONV, and HC groups, suggesting potential transcriptional reprogramming in LTCS (**Figure 3C**). Given the NK-cell dysregulation identified in our flow cytometry data, we specifically focused on NK populations and their progenitors to explore innate immune alterations. Notably, NK I cells (cluster C4) were significantly reduced in LTCS compared with CONV individuals (**Figure 3D**).

In addition, we annotated three further subclusters with altered abundance in LTCS: CD4^-^CD8^-^ double-negative (DN) T cells (C6), which were significantly upregulated in LTCS compared with HC; intermediate B cells (C21), which were decreased in LTCS compared with CONV (not significant); and memory B cells (C16), which were significantly upregulated in LTCS compared with HC (**Figure 3D**). Collectively, these findings confirm a reduction in NK I cells alongside selective remodeling of T- and B-cell subsets in LTCS, supporting a broad immune reorganization underlying the LTCS phenotype.

### NK cells are able to sense the pathological features of LTCS at the molecular level

**Figure 4:**
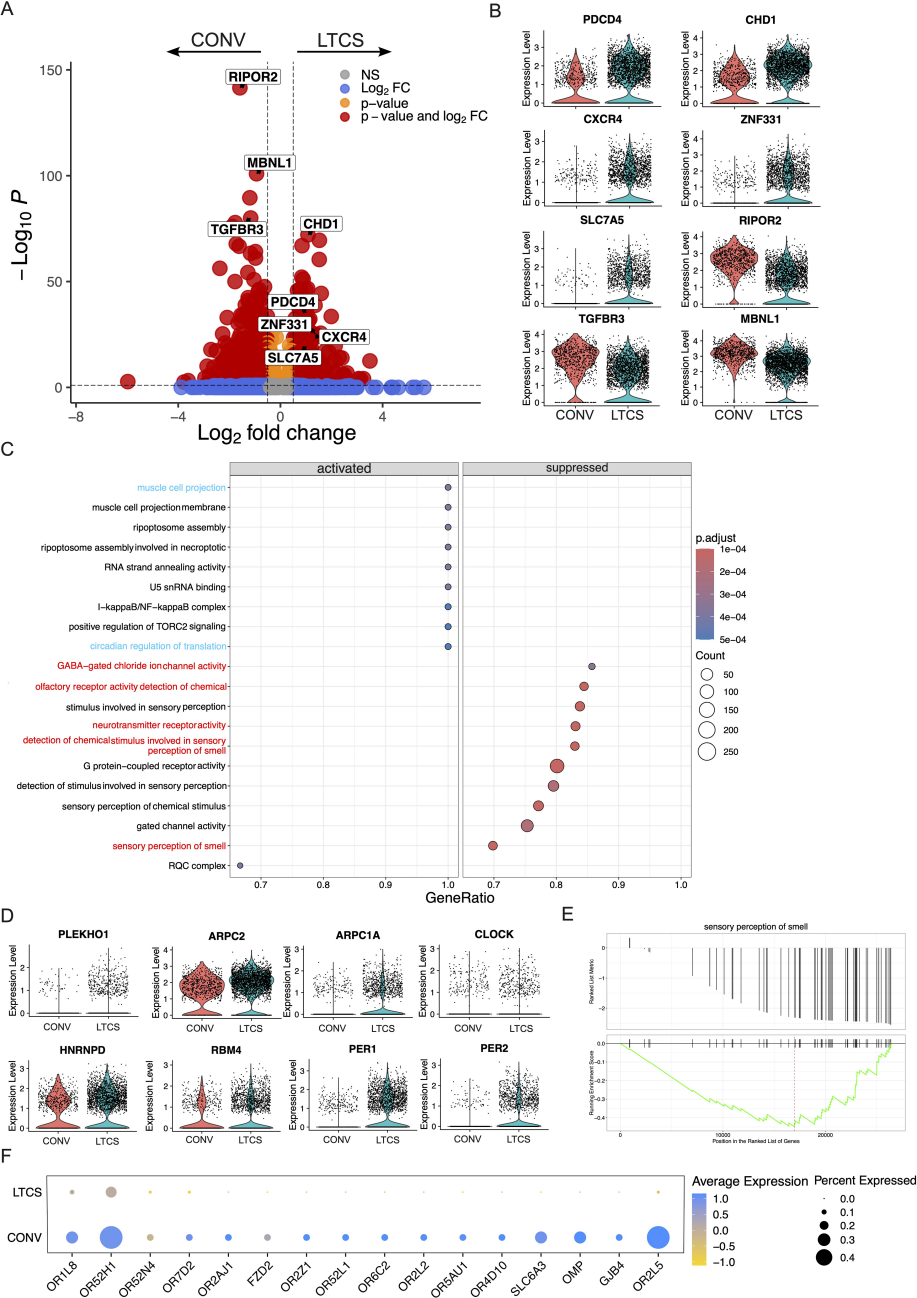
Dysregulated NK-cell gene expression and decreased signaling pathways related to smell, taste, and neurotransmitter sensing in LTCS patients. **(A)** Volcano plots demonstrate the significantly up- and downregulated genes in LTCS (*N* = 32) and convalescent (*N* = 6) patients with selected highlighted genes. The color-coded scheme shows the significance and log_2_ FC value. **(B)** Violin plots show the significantly up- (*PDCD4, CXCR4, CHD1, ZNF331, SLC7A5*) and downregulated (*RIPOR2, TGFBR3, MBNL1*) genes in LTCS patients and convalescents. **(C)** Dot plots show the significantly activated and suppressed pathways in the LTCS patient group compared with the convalescent group based on the gene set enrichment analysis. Dot size shows the count of genes in a specific pathway, and the red color shows the higher significance of the pathway. Muscle cell projection and circadian regulation of translation pathways were activated, while GABA-gated chloride ion channel, olfactory receptor activity detection of chemical stimulus involved in sensory perception, neurotransmitter receptor activity, and sensory perception of smell pathway were significantly suppressed in NK cells of LTCS patients. **(D)** Genes involved in muscle cell projection and circadian regulation of translation pathways. **(E)** Sensory perception of smell pathway. **(F)** Selected genes involved in the sensory perception of smell pathway.

NK cells are critical for maintaining inflammatory balance as part of the first line of defense within the innate immune system. They have a unique ability to balance host protection with tissue repair, particularly in mucosal tissues (63, 64). NK cells modulate immune responses following pathogen exposure, especially in the nasal and oral mucosa (65, 66). Our data, consistent with previous reports, show that NK-cell numbers are reduced during SARS-CoV-2 infection (67, 68). However, the role of NK cells in LTCS patients remains poorly defined. To gain deeper insight, we performed differential gene expression analysis of NK cells. We observed that 455 genes were significantly altered in LTCS compared with CONV individuals: 115 genes (25%), including *PDCD4*, *CHD1*, *CXCR4*, *ZNF331*, and *SLC7A5*, were upregulated, whereas 340 genes (75%), including *TGFBR3*, *RIPOR2*, and *MBNL1*, were downregulated (**Figures 4A, B**).

Interestingly, during chronic inflammation, neuroimmune crosstalk between the nervous and immune systems influences the behavior of olfactory stem cells and neuronal regeneration (69). Rather than promoting the regeneration of olfactory neurons, this interaction favors the maintenance of an epithelial-like, non-neuronal cell layer that provides immune protection—at the cost of proper neurosensory (smell) function (69). Consistent with this, persistent LTCS anosmia has been linked to immune-cell infiltration and altered gene expression within the olfactory epithelium (70).

To determine whether sensory and olfactory pathways are systemically dysregulated in LTCS, we performed GSEA. The GSEA revealed that muscle cell projection and circadian regulation of translation pathways were activated in LTCS patients, whereas GABA-gated chloride ion channel activity, olfactory receptor activity, neurotransmitter receptor activity, and chemical stimulus detection involved in sensory perception of smell were suppressed (**Figure 4C**). Furthermore, genes involved in muscle cell projection—*PLEKHO1*, *ARPC2*, and *ARPC1A*—were significantly upregulated in LTCS patients compared with CONV individuals (**Figure 4D**). Similarly, genes regulating circadian translation, including *CLOCK*, *HNRNPD*, *RBM4*, *PER1*, and *PER2*, were upregulated in LTCS patients (**Figure 4D**).

IL-4–IL-4Rα signaling has been shown to modulate neuronal–immune crosstalk, suggesting a functional link between olfactory impairment and neuroinflammation (71). One of the most common symptoms of LTCS is loss of smell (anosmia). Notably, genes related to sensory perception of smell—*Olfactory Receptor Family 1 Subfamily L Member 8* (*OR1L8*), *Olfactory Receptor Family 52 Subfamily H Member 1* (*OR52H1*), *Olfactory Marker Protein* (*OMP*), and *Olfactory Receptor Family 2 Subfamily L Member 5* (*OR2L5*)—were downregulated in LTCS patients (**Figure 4E, F**). Additionally, the dopamine transporter gene *SLC6A3*, which has been associated with several neuropsychiatric disorders (72) and sensory perception of smell (73), was significantly reduced in LTCS compared with CONV individuals (**Figure 4F**).

Similarly, when comparing HC and LTCS groups, the results mirrored those observed for CONV. Pathways related to olfactory receptor activity, chemical stimulus detection, fatty acid elongation, and saturated fatty acid metabolism were downregulated in LTCS relative to HC (**Supplementary Figure 5a**). It is likely that NK-cell lipid-sensing mechanisms are disrupted in LTCS, and identifying the specific lipid molecules involved may help to elucidate the underlying defects in NK-cell function. In contrast, pathways involving histone H3K9me/H3K9me2 demethylase activity and circadian regulation of translation were activated in LTCS patients compared with HC (**Supplementary Figure 5a**). Collectively, these GSEA results demonstrate that multiple molecular processes, including neurosensory signaling, lipid metabolism, and circadian regulation, are dysregulated in LTCS patients (**Supplementary Figures 5b, c**).

### Deranged cytotoxic, metabolic, and cytokine function of NK cells in LTCS

Our GSEA analysis revealed that sensory and chemical stimulus-related pathways were altered in NK cells. Therefore, we next examined genes associated with cytotoxicity, metabolism, and cytokine function. We found that most genes involved in cytotoxic activity were downregulated in LTCS patients compared with CONV individuals (**Supplementary Figure 6a**), whereas genes linked to autophagy, DNA metabolism, and NK-cell activation were upregulated. In addition, metabolic pathways were enriched in LTCS patients, while inflammation-sensing genes were expressed at lower levels (**Supplementary Figures 6a, b**).

To validate these findings at the protein level, we assessed perforin, granzyme B, and TNF-α expression in NK cells using flow cytometry. Perforin and granzyme B levels did not differ significantly between LTCS patients (*N* = 5) and HC (*N* = 5) individuals (**Supplementary Figure 6c**). However, the frequency of TNF-α-producing NK cells was reduced in LTCS patients. Taken together, these results indicate that NK cells in LTCS patients exhibit functional inpairment/exhaustion, characterized by altered transcriptional programs and reduced inflammatory cytokine production.

## Discussion

SARS-CoV-2 infection is able to persistently dysfunction several physiological systems, although the mechanisms leading to this debilitating condition remain unclear and thus an active area of research. Previous studies have reported heterogeneous immunological and inflammatory signatures in LTCS patients, with some describing elevated pro-inflammatory cytokines such as IFN-β, IFN-α, CXCL9, CXCL10, IFN-γ, CXCL6, and IL-8 (31, 51, 74, 75), while others identified downregulation of mediators including IL-18, MCP-1, TNF-RII, and TRAF2 (39, 52, 75). Thus, this conundrum highlights the possibility that distinct immunopathological trajectories exist among LTCS patients, potentially reflecting heterogeneity in host genetics, infection severity, viral persistence, or immune priming.

In our study, both pro-inflammatory cytokines (IFN-γ, TNF-α, IL-6, CXCL8 (IL-8), IL-7, IFN-α2) and the anti-inflammatory cytokine IL-10 were reduced in LTCS patients relative to healthy individuals. This suggests a dampened cytokine signaling state, rather than the persistent hyperinflammation often assumed in LTCS. We propose that this pattern resembles a long-term “refractory” phase, akin to neuronal hyperpolarization after action potential firing, in which the immune system remains suppressed below baseline activation thresholds. Such a prolonged hyporesponsive state could underlie many of the chronic manifestations of LTCS, though why some individuals recover more quickly remains unresolved. Immune exhaustion, particularly in NK and T cells, represents another plausible explanation, mirroring the well-characterized T-cell exhaustion observed in chronic viral infections (2, 29, 31, 33).

### T- and NK-cell alterations

Our data indicate that several immune alterations observed in LTCS reflect persistent post-infection changes, whereas others appear to be LTCS-specific features that may be linked to impaired recovery and chronic immune dysregulation. First, immune differences shared between CONV and LTCS relative to HC likely represent LTCS immune remodeling. CD56^+^CD16^+^ NK cells and CD56^+^CD3^+^ NKT cells were significantly reduced in LTCS compared with HC, but not when compared with CONV, suggesting that these reductions are established following SARS-CoV-2 infection and persist beyond the acute phase rather than being unique to LTCS. Similarly, the increased frequency of total CD3^+^ T cells in LTCS relative to HC reflects a broader shift in lymphocyte composition following infection. This is consistent with our scRNA-seq findings, which showed both reduced NK-cell frequencies and widespread transcriptional downregulation in these cells.

A previous study reported altered T-cell distributions in LTCS, including increased CD4^+^ T-cell frequencies, exhausted SARS-CoV-2-specific CD8^+^ T cells, and impaired coordination between T- and B-cell responses (41). Immune alterations that distinguish LTCS from CONV are more likely to reflect failure to resolve immune activation and may contribute to LTCS pathogenesis. Notably, CD16^-^CD14^-^ myeloid monocytes were significantly reduced in LTCS compared with CONV, indicating a divergence in innate immune compartment recovery between individuals who resolve infection and those who develop LTCS. Additionally, naive CD4^+^ T cells and CD4^+^HLA-DR^+^CCR7^+^ activated central memory T cells showed a downward trend in LTCS relative to CONV, suggesting impaired replenishment or maintenance of key adaptive immune subsets in LTCS. While these changes did not reach statistical significance, their consistent directionality supports a model of disrupted immune homeostasis in LTCS. Furthermore, activated T-cell subsets such as CD4^+^CD38^+^CCR7^+^ T cells were significantly elevated in CONV compared with HC but not in LTCS, suggesting that CONV individuals mount a transient, resolving adaptive immune response that is blunted or dysregulated in LTCS. Similarly, CD8^+^CD38^+^CCR7^+^ and CD8^+^HLA-DR^+^CCR7^+^ T cells were reduced in CONV relative to HC, with a non-significant upward trend in LTCS, highlighting altered kinetics of T-cell activation and resolution between the two post-infection outcomes. Interestingly, CXCR4, CXCR5, and CCR6 expression—reported previously as enriched in SARS-CoV-2-specific CD4^+^ T cells (41)—was also highly expressed in NK cells in our LTCS cohort. This suggests potential trafficking of both T and NK cells from the blood to inflamed tissues, which may explain their reduced peripheral abundance and contribute to ongoing pathology. Taken together, these stratified comparisons support a model in which LTCS is characterized not merely by persistent immune alterations following SARS-CoV-2 infection, but by selective failures in innate–adaptive immune coordination and immune recovery.

### Molecular and metabolic reprogramming in NK cells

At the transcriptional level based on scRNA-seq, nearly two-thirds of NK-cell genes were downregulated in LTCS patients, again underscoring their suppressed state. Upregulated genes, including PDCD4, CHD1, CXCR4, ZNF331, and SLC7A5, have all been linked to immune dysregulation or disease pathology. For example, PDCD4 has been implicated in impaired autophagy and α-synuclein accumulation in Parkinson’s disease models (76), which resonates with the high prevalence of neurological symptoms in our LTCS cohort and the reported increased risk of neurodegenerative disease post-COVID-19 (77). CHD1 influences IL-6 transcription and recruitment of myeloid-derived suppressor cells (78), suggesting a role in immunosuppressive remodeling. SLC7A5 regulates amino acid transport and metabolic reprogramming in NK cells and monocytes (79, 80), and its upregulation may reflect an adaptive attempt to balance cytokine production with metabolic demand or residual viral antigens (81, 82). PDCD4 is involved in the regulation of metabolic and co-signaling pathways, indicating that it acts as a molecular regulator that could influence the transition from an effector state to an exhausted state under chronic stimulation.

### Neuroimmune and metabolic pathways linked to symptoms

Pathway enrichment analysis highlighted the suppression of neurotransmission and sensory perception networks (e.g., GABA-gated chloride channel activity, neurotransmitter receptor signaling, olfactory and gustatory receptor activity) in LTCS NK cells. These findings align with the common neurological, sensory, and neuropsychiatric symptoms of LTCS, including anosmia, dysgeusia, fatigue, and “brain fog” (38–41). Conversely, pathways involved in circadian translation regulation and muscle projection were upregulated, potentially contributing to sleep dysregulation and exercise intolerance (42–44).

A recent mechanistic study demonstrated that viral RNA-driven type I interferon activity reduces serotonin production in LTCS patients (17), consistent with our observation of reduced IFN-α2 and IFN-γ. Since IFN-γ is classified as a type II IFN rather than type I, the crosstalk between type I and type II IFNs during post-viral infection recovery could be altered in LTCS patients. Additionally, correlation analysis in LTCS patients among cytokines and amino acids revealed that histidine and glutamine were uniquely associated mainly with pro-inflammatory cytokines (44). Furthermore, triglycerides and apolipoproteins Apo-A1 and A2 in LTCS patients show COVID-19-like alterations compared with HC. LTCS and acute COVID-19 patients were separated by phenylalanine, 3-hydroxybutyrate, and glucose concentrations, pointing to an imbalanced energy metabolism; thus, we described an imbalance between cytokine, lipid, and metabolite levels in LTCS (44). Thus, speculation of potentially dysregulated serotonin metabolism and precision nutritional metabolic programming (83) may connect immune dysfunction with neurocognitive symptoms, reinforcing the importance of integrated immunometabolic profiling. However, further studies are warranted to link this hypothesis.

### Limitation of the study

Our study has several limitations. First, LTCS disproportionately affects women (81, 82), and our cohort reflected this bias (60% women), which recapitulates previously assessed parameters, thus verifying previously published findings. Furthermore, age is another confounding factor in our study, which is mostly dominated by male participants. We also acknowledge that “days post-infection” may be imprecise for some participants (e.g., based on symptom onset or test date); we therefore interpret timing-adjusted analyses as supportive/sensitivity analyses and note this limitation. While this mirrors epidemiological trends, gender-specific immune differences may confound interpretation and require larger stratified cohorts. Second, limited PBMC yields restricted parallel flow cytometry and scRNA-seq for some patients. However, the independent datasets provide mutual validation of key findings. Third, we analyzed samples at single time points; longitudinal profiling will be essential to capture dynamic immune remodeling and recovery trajectories. Future studies should integrate immune, metabolic, and neuroimaging approaches to decode the interplay between viral persistence, immune exhaustion, and neuroinflammation in long COVID. Understanding these pathways may enable stratification of patients into immunopathological endotypes and guide targeted interventions, ranging from cytokine modulation and checkpoint blockade to metabolic therapies.

## Data Availability

The datasets presented in this study can be found in online repositories. The names of the repository/repositories and accession number(s) can be found below: https://zenodo.org/uploads/14886569 and sequencing data available through GEO accession; GSE320507.

## Supplementary material

Supplementary Figure 1 Patient demographics. **(a)** Mean age of HC (N = 66), CONV (N = 35) and LTCS (N = 114). Kruskal Wallis test was used for statistical significance with Dunn’s *post-hoc* test for multiple comparisons amongst different groups. P value ≤ 0.05 is considered significant. **(b)** Gender based mean age of HC, CONV and LTCS patients. **(c)** Depiction of male and female distribution amongst different groups using pie charts. **(d)** Percentage symptoms of LTCS (N = 114) in the cohort. **(e)** Percentage symptoms in male and female LTCS patients.

Supplementary Figure 2 Cytokine levels in LTCS and convalescent patients. **(a)** Antibody levels (Nucleocapsid) were modeled as a function of days post-infection and cohort using linear regression, including a cohort × time interaction term to assess whether antibody kinetics differed between LTCS patients (N = 94) and CONV (N = 24) participants. Sensitivity analyses restricting samples to ≤200 days post-infection yielded consistent results, and no significant difference was observed between LTCS patients and CONV participants. **(b)** CXCL8 (IL-8) and IL-7 were significantly downregulated in LTCS (N=86) patients compared to HC (N=66) group. Low IFN-α2 and high IL-1RA levels were detected in LTCS patients compared with HC respectively, however, no difference was observed amongst both the group. CXCL-10 (IP-10) levels were significantly reduced in LTCS compared with CONV. P value ≤ 0.05 is considered significant. **(c)** The other cytokines and chemokines such as G-CSF, MCP-1, MIP-1α, IL-2, G-CSF, and MCP-1 were also detected in LTCS patients, however, no difference was observed amongst all different groups. P value ≤ 0.05 is considered significant.

Supplementary Figure 3 Gating strategy for immunophenotyping of PBMCs from LTCS patients. First stained cells were plotted against SSC-A *vs* time to identify the based on FlowCutBad events using machine learning program from FlowJo. We detected minimal amounts of bad events in the samples. Later, we gated out debris and then we detected singlets based on FSC-A *vs* FSC-H. These singlets were further used for the estimation of lymphocytes and monocyte cell populations based on arbitrary gate on cell size and granularity. Firstly, monocytes were plotted against CD14 and CD16 followed by CD16^+^CD14^-^ monocytes for activation markers HLA-DR and CD38. Secondly, lymphocyte population was plotted against CD3 and CD19 to identify three subsets – namely CD3^+^ T cells, CD19^+^ B cells and CD19^-^CD3^-^ myeloid cells. We started to identify the activation status of CD19^+^ B cells by gating against HLA-DR and CD38. Furthermore, we followed the CD19^-^CD3^-^ myeloid cells to identify the NK cell subsets. We gated CD19^-^CD3^-^ myeloid cells against HLA-DR *vs* CD56 and used CD56^+^HLA-DR^-^ cells for NK cell subsets by plotting against CD56 *vs* CD16. We identified three major NK cell subsets – NKI; classical NK cells based on CD56^+^CD16^+^, NKII; early NK cells based CD56^high^C16^-^, NKIII; terminal NK cells based CD56^-^CD16^+^. Later, CD56^+^HLA-DR^-^ cells were examined for dual expression of CD56 *vs* CD8, CD69, CD38, CD45RA, CCR7, CD154. Further, we followed CD3^+^ T cells for NKT, CD4 and CD8 cell subsets, therefore, we first gated CD3^+^ T cells against CD56 *vs* either CD4 or CD8 to identify NKT cells. NKT cell further examined for the expression of CCR7, CD69, CD45RA, HLA-DR, and CD16. CD3^+^CD56^-^ T cells were used for quantification of CD4 and CD8 cell subsets and plots against CD8 *vs* CD4. CD4^+^ T cells were gated against FOXP3 to identify Tregs. Later, CD4^+^FOXP3^-^ T cells were gated against CD45RA *vs* CCR7 followed by CCR7 *vs* HLA-DR and CD38. Similarly, CD8^+^ T cells were gated against CD45RA *vs* CCR7 followed by CCR7 *vs* HLA-DR and CD38.

Supplementary Figure 4 Quality control, integration, doublet removal and cell identification. **(a)** Violin plots show data pre- and post-processing. **(b)** UMAP plot shows the pre- and post-integration (CCA) of all the samples including the doublet and singlet cells. Color coded information for HC, CONV and LTCS is given for individual plots. **(c)** Identification of cell clusters based on RNA based gene clusters and canonical markers of lymphocytes and monocytes. **(d)** Heatmap shows top 10 genes in each cluster. Cluster 9 (Doublet) has mixed features thus, eliminated from further analysis. **(e)** Heatmap shows cluster 9 contains doublet cells. **(f)** UMAP plots for individual gene markers for different cell types.

Supplementary Figure 5 Dysregulated NK cells pathways in LTCS patients compared with HC. **(a)** Dot plots show the significantly activated and suppressed pathways in LTCS (N = 32) patient group compared with HC (N = 8) group based on gene set enrichment analysis. Dot size show count of genes in a specific pathway and red color show the higher significance of the pathway. Histone H3K9me/H3K9me2 demethylase activity and circadian regulation of translation pathways were activated whilst GABA-gated chloride ion channel, olfactory receptor activity detection of chemical, stimulus involved in sensory perception, neurotransmitter receptor activity, and sensory perception of smell pathways were significantly suppressed in NK cells of LTCS patients. **(b)** Suppressed pathways – olfactory receptor activity, neurotransmitter and sensor perception of taste. **(c)** Activated pathways – histone H3K9me/H3K9me2 demethylase activity and viral translational termination-reinitiation.

Supplementary Figure 6 Gene expression analysis of NK cells for their cytotoxic, metabolic and cytokine sensing function. **(a)** Dot plot shows gene expression in NK cells for LTCS, CON and HC groups for their cytotoxic, metabolic and cytokine sensing function. **(b)** Key cytokines - TNF, IL-18, IFNG and IL-32 gene expression analysis which are involved in modulation of NK cell function. **(c)** TNF-α, granzyme B and perforin expression in NK cells obtained from HC (N = 6) and LTCS (N = 6) patients. FACS plots representing perforin and TNF-α staining for HC and LTCS patients (Left side). Bar-plot represents the TNF-α expressing NK cells from HC and LTCS patients. P value ≤ 0.05 is considered significant.
