# Supplementary figures and images for: Dysregulated NK-cell gene expression defines the enduring symptoms of long COVID-19
Source: Front Immunol. 2026 Mar 9;17:1720551. doi: 10.3389/fimmu.2026.1720551 (PMC13006230; doi:10.3389/fimmu.2026.1720551)

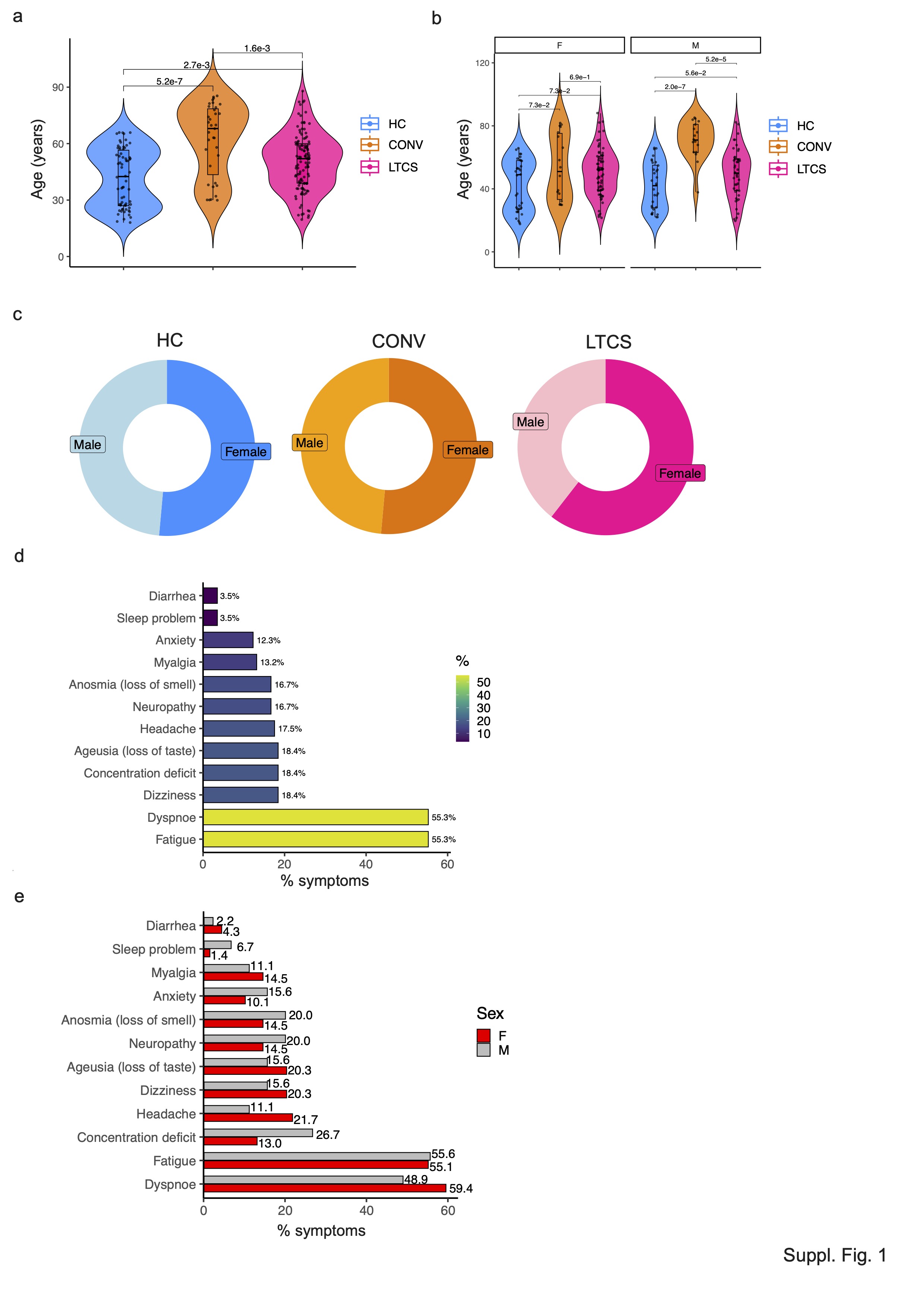

Supplement: Supplementary Figure 1 — Patient demographics. (a) Mean age of HC (N = 66), CONV (N = 35) and LTCS (N = 114). Kruskal Wallis test was used for statistical significance with Dunn’s post-hoc test for multiple comparisons amongst different groups. P value ≤ 0.05 is considered significant. (b) Gender based mean age of HC, CONV and LTCS patients. (c) Depiction of male and female distribution amongst different groups using pie charts. (d) Percentage symptoms of LTCS (N = 114) in the cohort. (e) Percentage symptoms in male and female LTCS patients. [file Image1.jpeg]

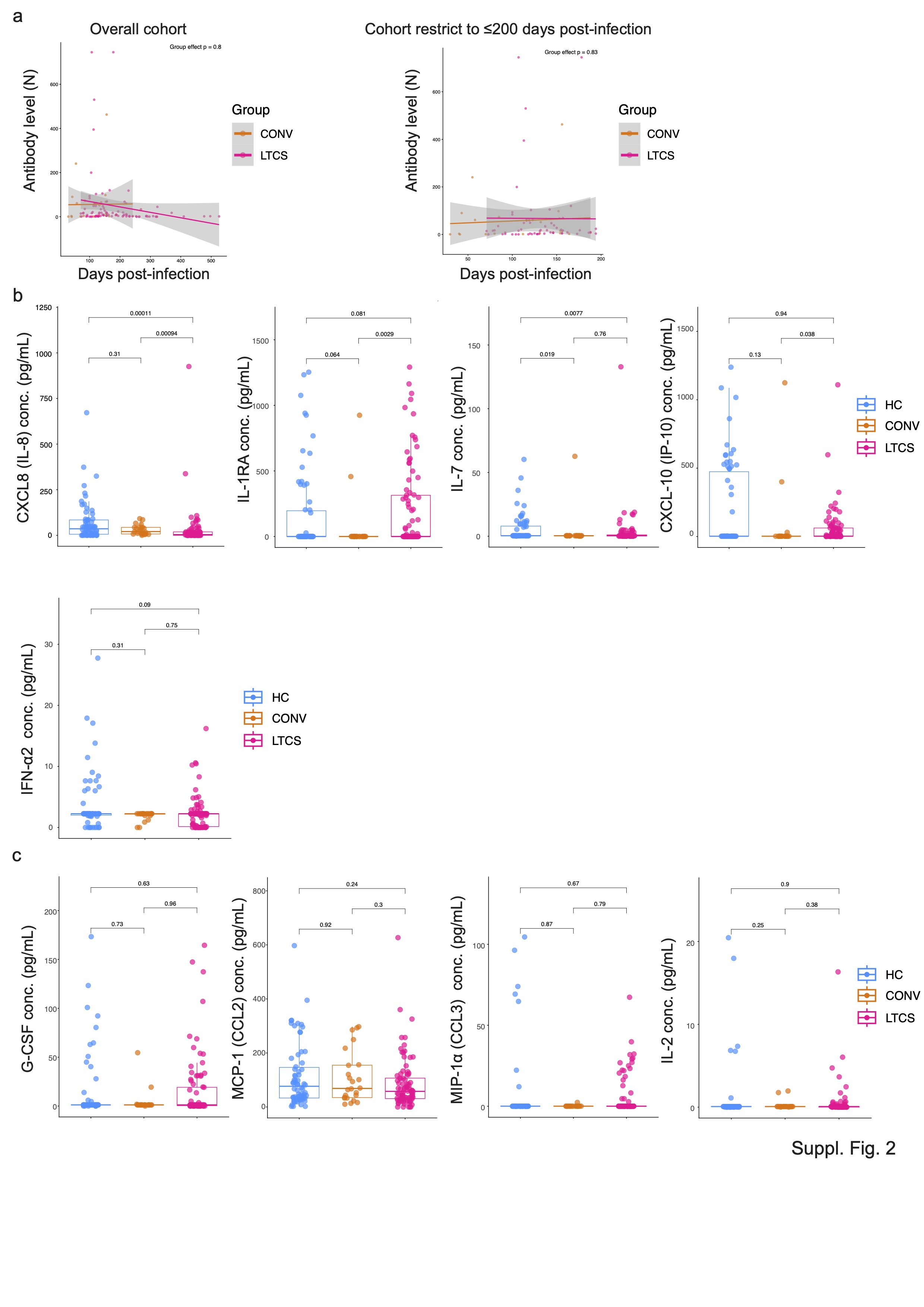

Supplement: Supplementary Figure 2 — Cytokine levels in LTCS and convalescent patients. (a) Antibody levels (Nucleocapsid) were modeled as a function of days post-infection and cohort using linear regression, including a cohort × time interaction term to assess whether antibody kinetics differed between LTCS patients (N = 94) and CONV (N = 24) participants. Sensitivity analyses restricting samples to ≤200 days post-infection yielded consistent results, and no significant difference was observed between LTCS patients and CONV participants. (b) CXCL8 (IL-8) and IL-7 were significantly downregulated in LTCS (N=86) patients compared to HC (N=66) group. Low IFN-α2 and high IL-1RA levels were detected in LTCS patients compared with HC respectively, however, no difference was observed amongst both the group. CXCL-10 (IP-10) levels were significantly reduced in LTCS compared with CONV. P value ≤ 0.05 is considered significant. (c) The other cytokines and chemokines such as G-CSF, MCP-1, MIP-1α, IL-2, G-CSF, and MCP-1 were also detected in LTCS patients, however, no difference was observed amongst all different groups. P value ≤ 0.05 is considered significant. [file Image2.jpeg]

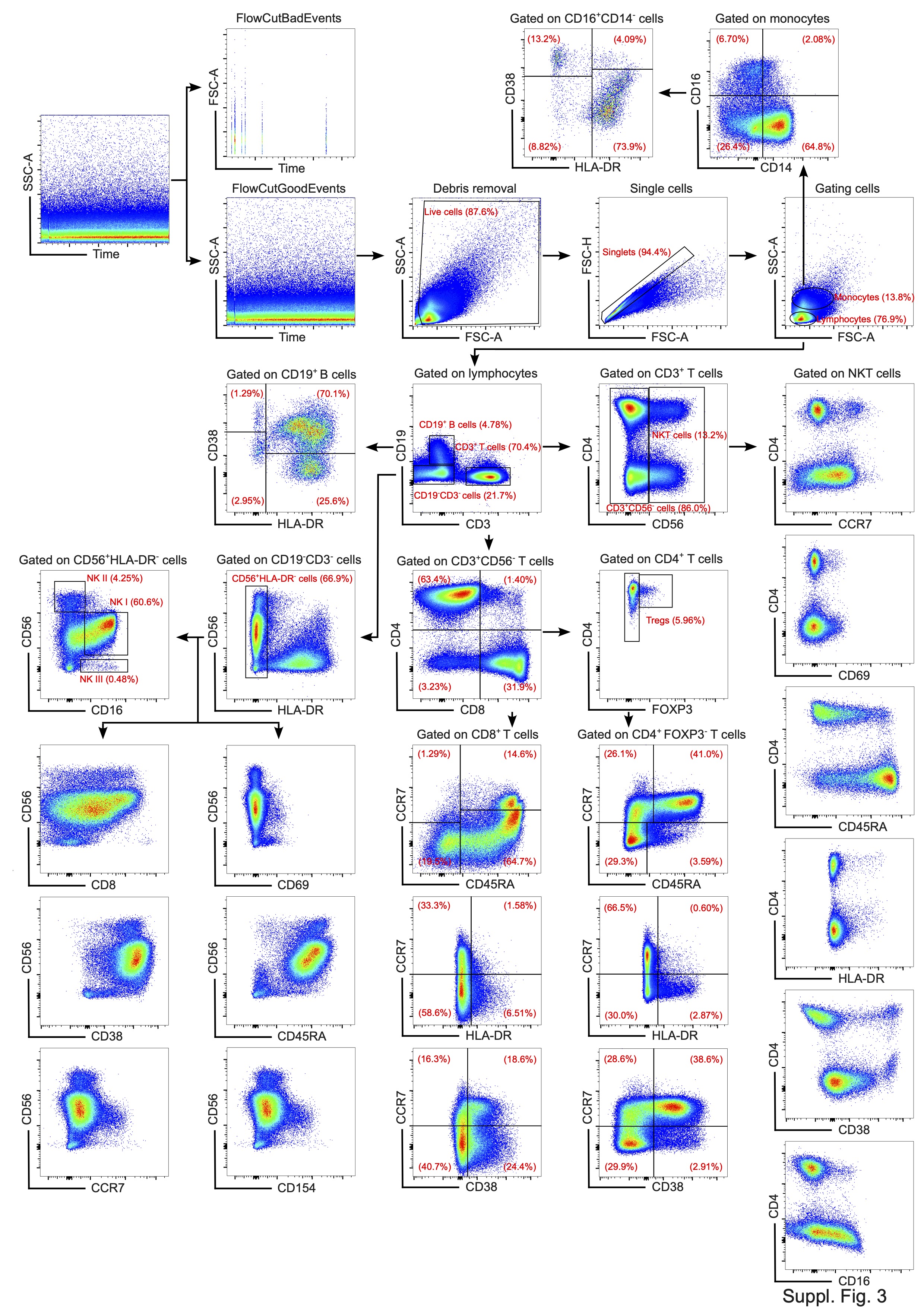

Supplement: Supplementary Figure 3 — Gating strategy for immunophenotyping of PBMCs from LTCS patients. First stained cells were plotted against SSC-A vs time to identify the based on FlowCutBad events using machine learning program from FlowJo. We detected minimal amounts of bad events in the samples. Later, we gated out debris and then we detected singlets based on FSC-A vs FSC-H. These singlets were further used for the estimation of lymphocytes and monocyte cell populations based on arbitrary gate on cell size and granularity. Firstly, monocytes were plotted against CD14 and CD16 followed by CD16+CD14- monocytes for activation markers HLA-DR and CD38. Secondly, lymphocyte population was plotted against CD3 and CD19 to identify three subsets – namely CD3+ T cells, CD19+ B cells and CD19-CD3- myeloid cells. We started to identify the activation status of CD19+ B cells by gating against HLA-DR and CD38. Furthermore, we followed the CD19-CD3- myeloid cells to identify the NK cell subsets. We gated CD19-CD3- myeloid cells against HLA-DR vs CD56 and used CD56+HLA-DR- cells for NK cell subsets by plotting against CD56 vs CD16. We identified three major NK cell subsets – NKI; classical NK cells based on CD56+CD16+, NKII; early NK cells based CD56highC16-, NKIII; terminal NK cells based CD56-CD16+. Later, CD56+HLA-DR- cells were examined for dual expression of CD56 vs CD8, CD69, CD38, CD45RA, CCR7, CD154. Further, we followed CD3+ T cells for NKT, CD4 and CD8 cell subsets, therefore, we first gated CD3+ T cells against CD56 vs either CD4 or CD8 to identify NKT cells. NKT cell further examined for the expression of CCR7, CD69, CD45RA, HLA-DR, and CD16. CD3+CD56- T cells were used for quantification of CD4 and CD8 cell subsets and plots against CD8 vs CD4. CD4+ T cells were gated against FOXP3 to identify Tregs. Later, CD4+FOXP3- T cells were gated against CD45RA vs CCR7 followed by CCR7 vs HLA-DR and CD38. Similarly, CD8+ T cells were gated against CD45RA vs CCR7 followed by CCR7 vs HLA-DR and CD38. [file Image3.jpeg]

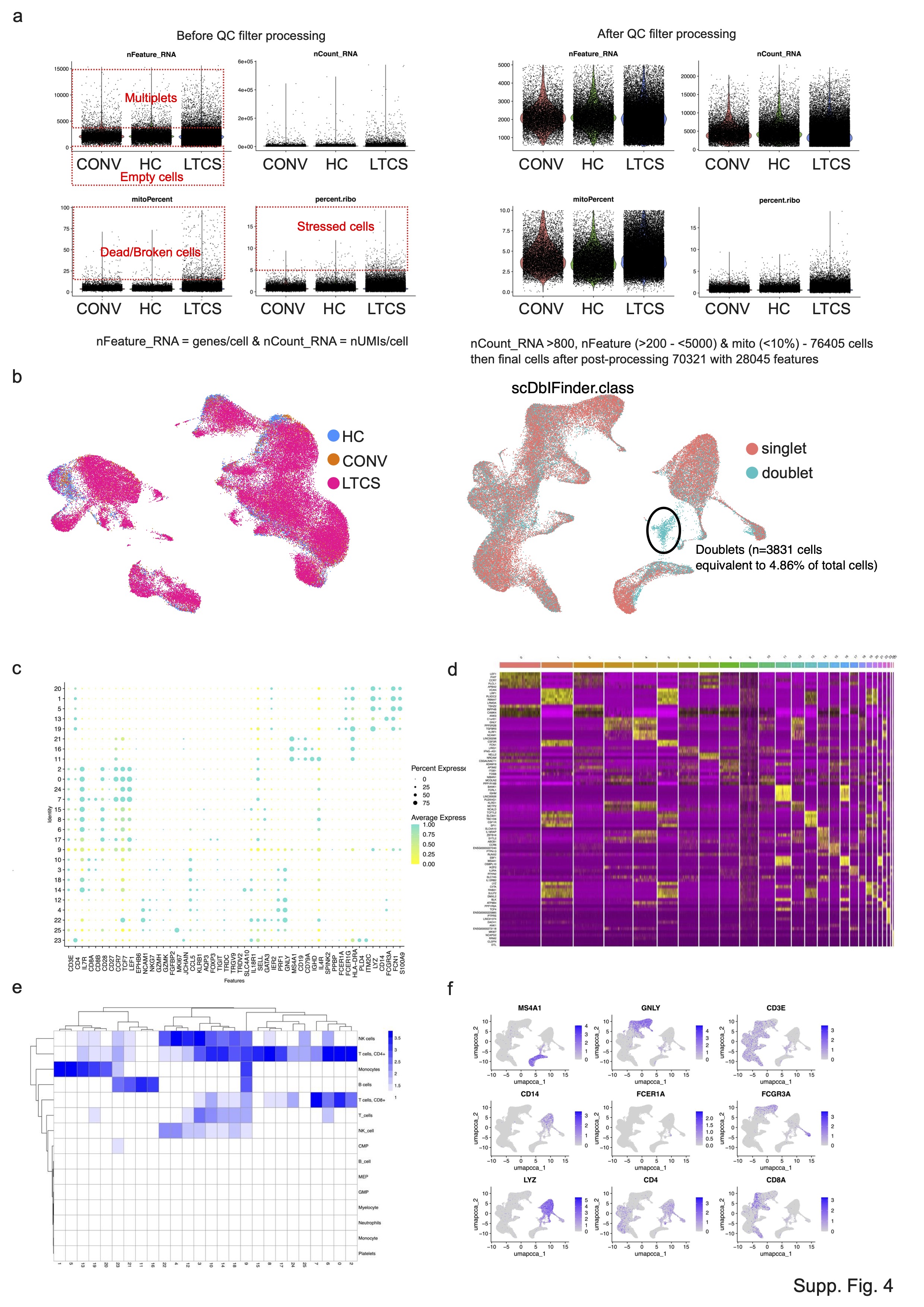

Supplement: Supplementary Figure 4 — Quality control, integration, doublet removal and cell identification. (a) Violin plots show data pre- and post-processing. (b) UMAP plot shows the pre- and post-integration (CCA) of all the samples including the doublet and singlet cells. Color coded information for HC, CONV and LTCS is given for individual plots. (c) Identification of cell clusters based on RNA based gene clusters and canonical markers of lymphocytes and monocytes. (d) Heatmap shows top 10 genes in each cluster. Cluster 9 (Doublet) has mixed features thus, eliminated from further analysis. (e) Heatmap shows cluster 9 contains doublet cells. (f) UMAP plots for individual gene markers for different cell types. [file Image4.jpeg]

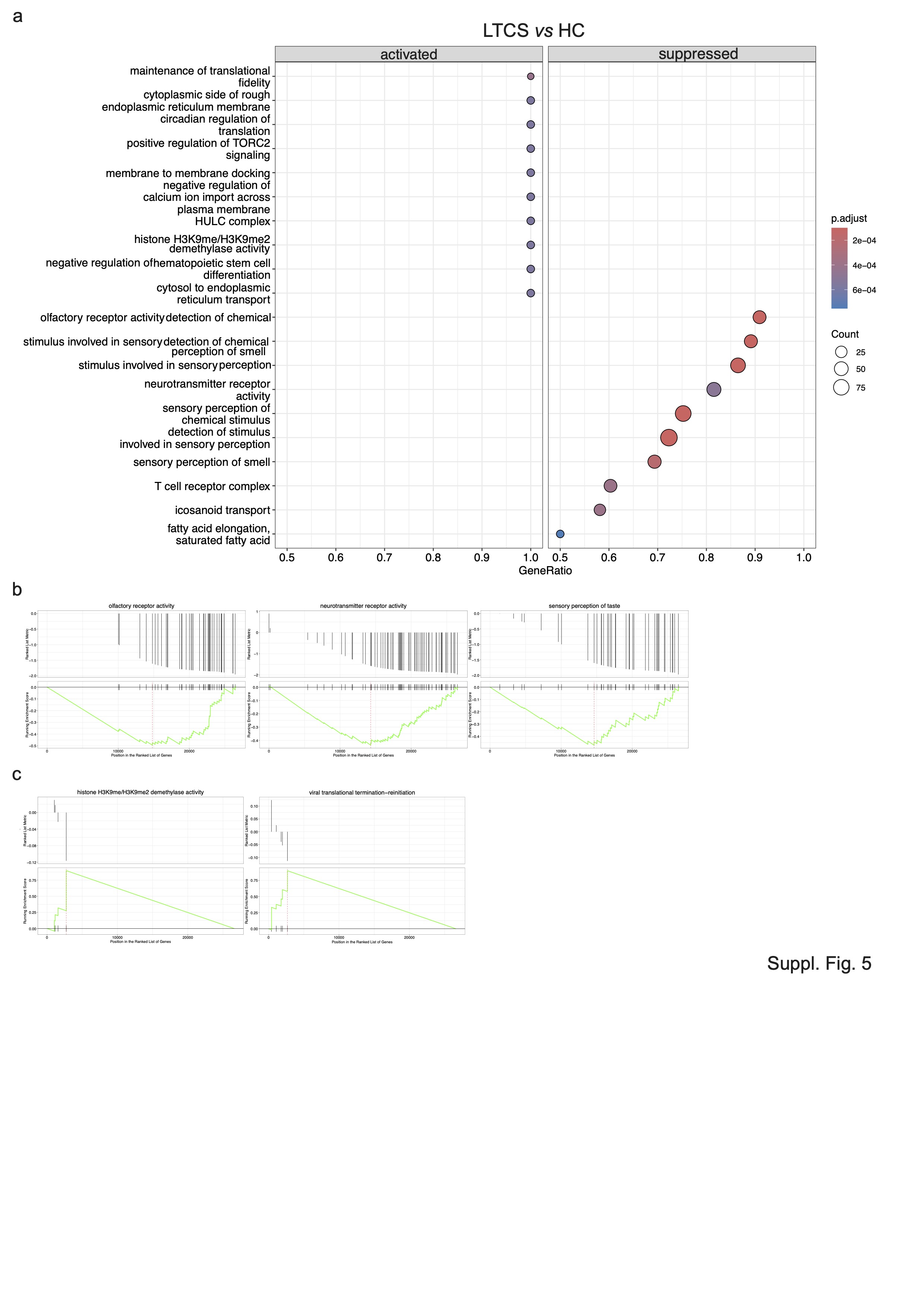

Supplement: Supplementary Figure 5 — Dysregulated NK cells pathways in LTCS patients compared with HC. (a) Dot plots show the significantly activated and suppressed pathways in LTCS (N = 32) patient group compared with HC (N = 8) group based on gene set enrichment analysis. Dot size show count of genes in a specific pathway and red color show the higher significance of the pathway. Histone H3K9me/H3K9me2 demethylase activity and circadian regulation of translation pathways were activated whilst GABA-gated chloride ion channel, olfactory receptor activity detection of chemical, stimulus involved in sensory perception, neurotransmitter receptor activity, and sensory perception of smell pathways were significantly suppressed in NK cells of LTCS patients. (b) Suppressed pathways – olfactory receptor activity, neurotransmitter and sensor perception of taste. (c) Activated pathways – histone H3K9me/H3K9me2 demethylase activity and viral translational termination-reinitiation. [file Image5.jpeg]

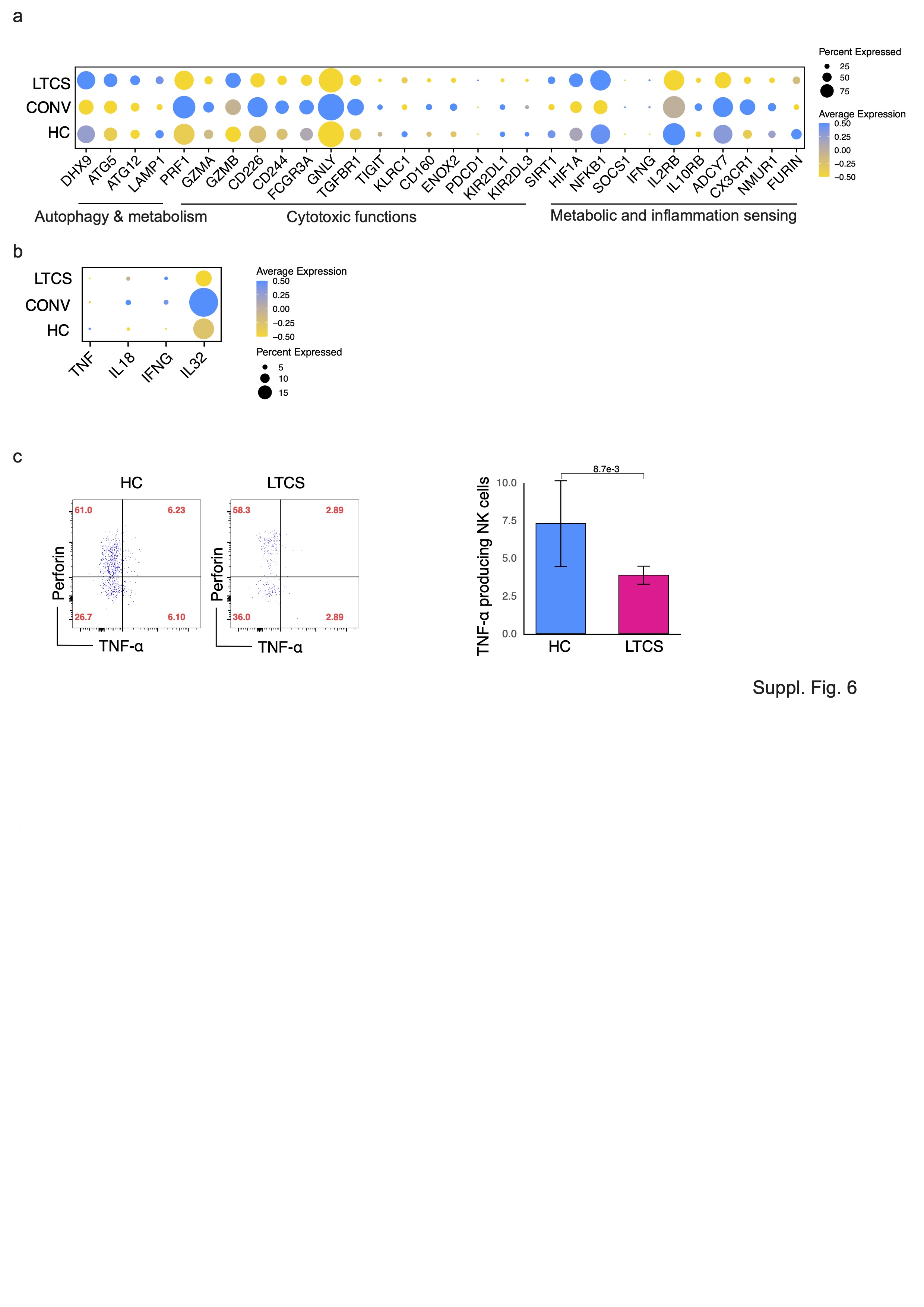

Supplement: Supplementary Figure 6 — Gene expression analysis of NK cells for their cytotoxic, metabolic and cytokine sensing function. (a) Dot plot shows gene expression in NK cells for LTCS, CON and HC groups for their cytotoxic, metabolic and cytokine sensing function. (b) Key cytokines - TNF, IL-18, IFNG and IL-32 gene expression analysis which are involved in modulation of NK cell function. (c) TNF-α, granzyme B and perforin expression in NK cells obtained from HC (N = 6) and LTCS (N = 6) patients. FACS plots representing perforin and TNF-α staining for HC and LTCS patients (Left side). Bar-plot represents the TNF-α expressing NK cells from HC and LTCS patients. P value ≤ 0.05 is considered significant. [file Image6.jpeg]
